# Circulating Tumor DNA (ctDNA) for Minimal Residual Disease (MRD) Detection in Colorectal Cancer (CRC): Current Evidence, Clinical Applications, and Future Directions

**DOI:** 10.3390/curroncol33100606

**Published:** 2026-10-09

**Authors:** Hannah Hyde, Emma Mageau, Chelsea M. Leduc, Mitul J. Madhu, Noah Dickinson, Tafheem-Un Nisa, Karolina M. Czajka, Stacey A. Santi, Prashant Jani, Grace W. Ma, Sujeenthar Tharmalingam

**Affiliations:** 1 Medical Sciences Division, NOSM University, 935 Ramsey Lake Rd., Sudbury, ON P3E 2C6, Canadammadhu@laurentian.ca (M.J.M.); kczajka@nosm.ca (K.M.C.);; 2 School of Natural Sciences, Laurentian University, Sudbury, ON P3E 2C6, Canada; 3 Health Sciences North Research Institute, Sudbury, ON P3E 2H2, Canada; 4 Health Sciences North, Sudbury, ON P3E 2H2, Canada; 5 Clinical Sciences Division, NOSM University, 935 Ramsey Lake Rd., Sudbury, ON P3E 2C6, Canada

**Keywords:** colorectal cancer (CRC), colon cancer, rectal cancer, circulating tumor DNA (ctDNA), minimal residual disease (MRD), liquid biopsy, recurrence prediction, disease surveillance, tumor-informed assays, precision oncology

## Abstract

Minimal residual disease (MRD) is a major contributor to colorectal cancer (CRC) recurrence following curative-intent treatment, yet conventional surveillance methods may fail to detect residual disease at its earliest stages. Circulating tumor DNA (ctDNA) has emerged as a minimally invasive biomarker for MRD detection and postoperative monitoring. We conducted a structured PubMed-based literature review and included 94 publications evaluating ctDNA in CRC. Across the literature, postoperative ctDNA positivity was consistently associated with increased recurrence risk and poorer disease-free and overall survival, while serial monitoring improved detection sensitivity and enabled assessment of treatment response. ctDNA frequently identified molecular recurrence approximately 2.5–11.5 months before conventional surveillance. Both tumor-informed and tumor-naive strategies were represented, using PCR/ddPCR, targeted next-generation sequencing, whole-exome or whole-genome sequencing, methylation-based assays, and emerging multiomic approaches. Although ctDNA demonstrates strong prognostic value, evidence supporting treatment modification based solely on ctDNA status remains limited. Further prospective randomized trials, assay standardization, and consensus clinical guidelines are required before routine implementation. Overall, ctDNA is a promising tool for recurrence prediction, surveillance, and precision management of CRC.

## Simple Summary

Recurrence remains a major challenge after treatment intended to cure colorectal cancer, as small amounts of residual cancer may remain below the detection limits of imaging and routine blood tests. This review examined 94 publications evaluating whether small fragments of genetic material shed by cancer cells into the blood can detect residual disease, predict recurrence, and monitor treatment response. Across the literature, detection of cancer-derived genetic material after treatment was consistently associated with a higher risk of recurrence and poorer survival. Repeated blood testing generally improved detection and often identified signs of returning cancer approximately 2.5 to 11.5 months before standard surveillance methods. The studies used a wide range of testing methods, blood collection schedules, and biological signals, which limited direct comparison. Test performance also varied with cancer stage, the biological characteristics of the tumor, and the site of recurrence. Although these blood tests are highly promising for risk assessment and surveillance, current evidence does not show that changing treatment solely on the basis of a positive result improves survival. Further clinical trials and standardized testing methods are needed before routine use, but these approaches could eventually support earlier recurrence detection, more personalized follow-up, and avoidance of unnecessary treatment.

## 1. Introduction

Colorectal cancer (CRC), encompassing malignancies of the colon and rectum, is one of the most common malignancies worldwide, accounting for approximately 9.6% of all new cancer diagnoses annually [1]. Globally, CRC is responsible for an estimated 865,000–903,000 deaths each year, highlighting its substantial impact on public health [2]. Despite advances in screening and treatment, patient outcomes remain highly dependent on disease stage at diagnosis. Five-year survival exceeds 90% for localized early-stage CRC but declines substantially with advancing disease, reaching approximately 10–15% for metastatic stage IV disease [3,4]. Several factors contribute to these poor outcomes, including late-stage diagnosis, treatment resistance, and limitations in current surveillance strategies for detecting recurrence at an early and treatable stage [5]. Despite undergoing curative-intent surgery, approximately 30% of CRC patients ultimately experience disease recurrence, suggesting the persistence of residual malignant cells following treatment [6].

Recent advances in molecular diagnostics and DNA sequencing technologies have led to the development of novel approaches for cancer detection and monitoring. Among these is the concept of minimal residual disease (MRD), which refers to residual malignant cells that persist following cancer therapy but remain undetectable using conventional clinical and radiologic surveillance methods [7,8]. Residual malignant cells may persist following treatment through incomplete eradication, cellular dormancy, or treatment-associated selection, ultimately contributing to subsequent disease recurrence [9].

Growing evidence indicates that MRD plays a critical role in disease recurrence and metastatic progression. Studies assessing MRD following tumor resection have demonstrated that detectable MRD is strongly associated with an increased risk of recurrence and worse clinical outcomes [6,7,10,11]. Consequently, patients with detectable MRD generally exhibit a worse prognosis than MRD-negative patients and may benefit from more intensive surveillance or additional therapeutic intervention [6,8]. Although MRD has emerged as a promising biomarker for predicting postsurgical recurrence, its integration into routine clinical practice remains limited. At present, there are no universally accepted standards or guidelines governing MRD detection and clinical application, largely due to the limited availability of prospective randomized controlled trials validating its utility in guiding patient management [9,11,12].

Circulating tumor DNA (ctDNA) represents a promising approach for the sensitive and non-invasive detection of MRD [6,9,13]. ctDNA is a subset of cell-free DNA (cfDNA) that is released into the circulation from tumor cells through biological processes including apoptosis, necrosis, and other forms of cellular turnover [6,7]. Unlike non-tumor-derived cfDNA, ctDNA contains tumor-specific genetic and epigenetic alterations that can be used to identify and monitor malignant disease [6,12,13]. The abundance of ctDNA can reflect tumor burden and disease activity, although ctDNA shedding varies substantially according to tumor biology, disease site, and clinical context [7]. Quantification of ctDNA can therefore provide valuable information regarding tumor burden, treatment response, and disease progression [7].

One of the major advantages of ctDNA is its short half-life, which ranges from minutes to a few hours, allowing ctDNA levels to reflect changes in disease status in near real time [10,14,15]. As such, ctDNA has emerged as a promising biomarker for assessing residual disease and monitoring recurrence following treatment [14]. In addition, ctDNA can be detected through minimally invasive liquid biopsy approaches, most commonly using blood samples, although it may also be identified in other bodily fluids including saliva, urine, and cerebrospinal fluid [6]. Compared with conventional tissue biopsies, liquid biopsies offer a less invasive means of monitoring disease while enabling repeated sampling throughout the course of patient management [9,12].

In the postoperative surveillance of CRC, patient monitoring relies primarily on imaging modalities such as computed tomography (CT) and magnetic resonance imaging (MRI), in conjunction with serum biomarkers including carcinoembryonic antigen (CEA) [14]. While these approaches remain the standard of care, they have limited sensitivity for detecting MRD and early recurrence. Consequently, recurrent disease may remain undetected until tumor burden reaches a level that is visible on imaging studies or endoscopic surveillance [6].

Recent advances in ctDNA analysis have substantially expanded the molecular approaches available for MRD detection. Contemporary assays can identify tumor-associated genetic and epigenetic signals, including somatic mutations, structural variants, copy-number alterations, DNA methylation patterns, and other molecular features. These biomarkers may be evaluated using tumor-informed strategies, in which patient-specific alterations identified from tumor tissue are subsequently tracked in plasma, or tumor-naive approaches that detect predefined genomic, epigenomic, or multiomic signatures without prior tumor sequencing. Such approaches enable longitudinal assessment of residual disease, treatment response, and recurrence, with multiple studies demonstrating molecular detection months before conventional radiographic surveillance.

The objective of this literature review is to critically evaluate the current evidence supporting ctDNA for MRD detection and post-treatment surveillance in CRC. This review examines the prognostic and clinical utility of ctDNA, compares tumor-informed and tumor-naive detection strategies and their underlying genomic, epigenomic, and multiomic technologies, and discusses current limitations, clinical implementation challenges, and future directions.

## 2. Methodology

A structured literature search was conducted using the PubMed database to identify peer-reviewed research articles relevant to ctDNA and MRD in CRC. A predefined search strategy and eligibility criteria were used to identify relevant publications. Search terms included: circulating tumor DNA (ctDNA), minimal residual disease (MRD), and colorectal cancer (CRC). The literature search was initiated in September 2025, with the final update completed in July 2026 using the same search syntax and eligibility criteria and with no restrictions on publication date. The specific search string used was: ((((ctDNA[Title/Abstract]) OR (“circulating tumor DNA”[Title/Abstract]) OR (“circulating tumour DNA”[Title/Abstract]) OR (cfDNA[Title/Abstract]) OR (“cell-free DNA”[Title/Abstract]) OR (“liquid biopsy”[Title/Abstract])) AND ((MRD[Title/Abstract]) OR (“minimal residual disease”[Title/Abstract]) OR (“molecular residual disease”[Title/Abstract])) AND ((“colorectal cancer”[Title/Abstract]) OR (CRC[Title/Abstract]) OR (“colon cancer”[Title/Abstract]) OR (“rectal cancer”[Title/Abstract]))) NOT (Review[Publication Type])).

**Figure 1:**
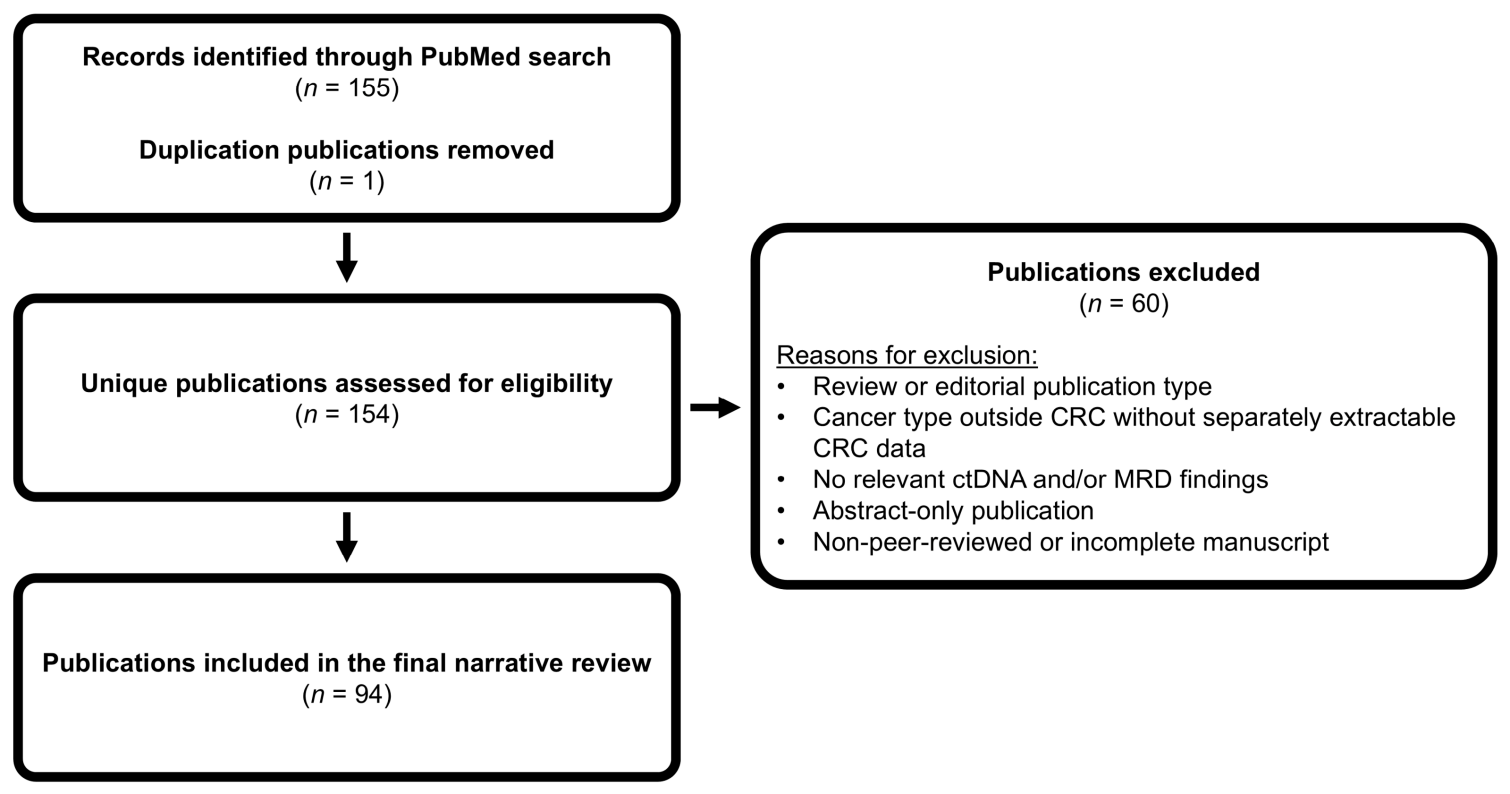
Flow diagram of publication selection for the literature review. The PubMed search identified 155 records. After removal of one duplicate publication, 154 unique records were assessed for eligibility. Sixty publications were excluded based on the predefined eligibility criteria, and 94 publications were included in the final narrative review.

A total of 155 records were identified through the search. One duplicate publication was removed, leaving 154 unique records for eligibility assessment. Publications were included if they: (1) investigated patients with CRC, including colon cancer, rectal cancer, or mixed CRC populations; (2) evaluated ctDNA or tumor-derived cfDNA in the context of MRD, recurrence detection, or post-treatment disease surveillance; and (3) reported original clinical data. Reviews, editorials, studies involving other cancer types without separately extractable CRC data, publications lacking relevant ctDNA or MRD findings, abstract-only reports, and non-peer-reviewed or incomplete manuscripts were excluded. Following eligibility assessment, 60 publications were excluded and 94 publications were included in the final review. The study selection process is summarized in Figure 1.

Relevant study characteristics and outcome measures were extracted and organized in Supplementary Table S1. Extracted information included publication details, study design or named trial/cohort, cancer site and disease stage, patient demographics, geographic location, cohort size, treatment context, assessment timepoint, ctDNA biomarkers, assay strategy, analytical platform, sensitivity, specificity, variant allele frequency or detection threshold, prognostic effect estimates including hazard ratios and 95% confidence intervals for recurrence-free, disease-free, and overall survival, recurrence rates, ctDNA clearance or persistence, clinical-utility endpoints, and other relevant clinical outcomes where reported. The extracted data were used to compare biomarker selection, assay design, analytical performance, and clinical applications across the included publications. Given the substantial heterogeneity in assay platforms and analytical methods, sampling timepoints and frequency, follow-up duration, disease stage and clinical setting, recurrence endpoints, and definitions or thresholds for MRD positivity, quantitative pooling was considered inappropriate. Findings were therefore synthesized narratively.

## 3. Results & Discussion

### 3.1. Study Characteristics

A total of 94 publications investigating the application of ctDNA for MRD detection in CRC were identified and included in this review (summarized in Supplementary Table S1). These publications encompassed a broad range of patient populations, study designs, and analytical approaches. Several publications were derived from shared or partially overlapping cohorts, particularly the CIRCULATE-Japan/GALAXY program. These reports were retained because they evaluated distinct clinical questions, assay platforms, patient subsets, or follow-up periods; however, their findings were synthesized collectively and were not treated as independent patient datasets. The evidence base ranged from individual case reports and small case series to large cohort studies involving more than 2000 patients. Case reports and very small series were interpreted descriptively rather than as comparative cohort evidence. Patient ages ranged from 17 to 96 years, and the included literature represented patients with colon cancer, rectal cancer, and mixed CRC populations.

The studies were conducted across multiple geographic regions, including Asia (Japan, China, Pakistan, Taiwan, Vietnam, and South Korea), Europe (Denmark, Italy, the Netherlands, the United Kingdom, Spain, Germany, Belgium, France, Austria, and Finland), Australia, and North America (Canada and the United States), reflecting the broad international interest in ctDNA-guided disease monitoring in CRC.

Considerable variability was observed in ctDNA detection methodologies. The included studies employed tumor-informed and tumor-naive assay strategies using a range of analytical platforms, including PCR- and ddPCR-based approaches, targeted and multiplex next-generation sequencing (NGS), whole-exome and whole-genome sequencing, methylation-based assays, and emerging multiomic and fragmentomic approaches. Commercially available platforms such as Signatera and Guardant Reveal were also frequently represented. Since many studies incorporated more than one analytical strategy or platform, these methodological categories were not mutually exclusive. This methodological diversity highlights the rapidly evolving nature of ctDNA-based MRD detection and underscores the need for continued evaluation and standardization of these approaches in CRC.

### 3.2. Analytical Characteristics, Clinical Performance, Prognostic Value, and Clinical Utility of ctDNA Assays

ctDNA studies report four related but conceptually distinct domains: analytical or operational characteristics, clinical assay performance, prognostic value, and clinical utility. Analytical characteristics describe the technical capability and workflow of an assay, including detection thresholds, variant allele frequency, error suppression, turnaround time, and tissue requirements. Clinical performance describes the ability of ctDNA testing at a defined clinical timepoint to identify molecular residual disease or predict recurrence and is commonly expressed using sensitivity, specificity, predictive values, and lead time. Prognostic value describes the association between ctDNA status or dynamics and subsequent outcomes, including recurrence-free survival, disease-free survival, overall survival, recurrence rates, and ctDNA clearance or persistence. Clinical utility evaluates whether using ctDNA results to guide management changes treatment use or improves patient outcomes. These measures should not be interpreted interchangeably. Table 1 summarizes the characteristics used to evaluate ctDNA assays for MRD detection in CRC for each domain separately.

Clinical performance varied considerably across studies because of differences in assay design, disease stage, tumor site, clinical endpoint, and the timing and frequency of sampling. In particular, sensitivity measured at a single postoperative landmark timepoint was often lower than sensitivity achieved through longitudinal monitoring. For example, one tumor-naive genomic and methylation-based assay demonstrated a sensitivity of 61.1% at the landmark timepoint and 83.3% with longitudinal assessment, with corresponding specificities of 87.9% and 89.5%, respectively [16]. Therefore, clinical sensitivity and specificity should be interpreted according to the assessment timepoint and endpoint, whereas analytical detection thresholds reflect technical assay capability rather than clinical recurrence-prediction performance. Similarly, hazard ratios, recurrence rates, and ctDNA-clearance outcomes should be interpreted according to the clinical endpoint, assessment timepoint, treatment context, and duration of follow-up and should not be interpreted as measures of assay sensitivity or specificity.

### 3.3. Existing Applications of ctDNA in Colorectal Cancer

The emergence of ctDNA as a promising biomarker for disease monitoring and MRD detection has provided clinicians with a minimally invasive tool for assessing tumor burden, treatment response, and relapse risk. Numerous clinical trials, cohort studies, and case reports have demonstrated its potential to inform postoperative surveillance and treatment decision-making in CRC.

Among the clinical applications of ctDNA monitoring, studies have observed that physicians are more likely to expedite treatment in post-surgical MRD-positive patients compared to MRD-negative or non-tested patients [6]. These treatment modifications may include the initiation of chemotherapy, extension of treatment duration, or adjustment of the chemotherapy regimen [17]. Conversely, patients deemed MRD-negative may undergo routine follow-up, potentially avoiding unnecessary chemotherapy and its associated toxicities [6]. As such, ctDNA provides a mechanism to personalize treatment strategies based on an individual patient’s risk of recurrence [6]. More recent prospective and randomized studies have further evaluated ctDNA-directed adjuvant treatment strategies, supporting the emerging role of MRD status in postoperative therapeutic decision-making [18,19].

This improved approach to patient management is strongly associated with clinical outcomes and prognosis. A growing body of literature specifically supports the utility of ctDNA monitoring and MRD detection in CRC. Given that ctDNA performance and clinical interpretation vary according to the timing of sample collection relative to treatment, findings are distinguished throughout this section as preoperative or baseline, postoperative landmark, post-neoadjuvant or post-adjuvant, and longitudinal surveillance assessments. Results obtained during these different clinical phases should not be interpreted interchangeably.

#### 3.3.1. Prognostic Value and Recurrence Prediction

Numerous studies have demonstrated that the presence of ctDNA following surgery possesses strong prognostic value for predicting cancer recurrence in CRC patients [15,20,21,22]. Multiple investigations have reported substantially higher recurrence rates among patients who remain ctDNA-positive after treatment. For example, one study demonstrated that detectable ctDNA following surgery was associated with a 79% risk of disease recurrence, compared to only 9.8% in ctDNA-negative patients [22]. More recently, Kalil et al. reported that 81% of patients with detectable ctDNA at the time of colorectal liver metastasis resection developed disease recurrence, compared with 46% of patients with undetectable ctDNA [21]. Similarly, Schøler et al. found that ctDNA negativity was associated with the absence of relapse during the study follow-up period, whereas all ctDNA-positive patients experienced disease recurrence [15]. In a comparable study, the three-year recurrence-free interval (RFI) was 77% in patients with undetectable ctDNA but only 30% in patients with detectable ctDNA [20].

One explanation for the prognostic value of ctDNA is that it reflects the presence of postsurgical MRD and may correlate with the burden of occult micrometastatic disease within the patient [22,23]. Consequently, detectable ctDNA may serve as a surrogate marker of residual malignant disease, often preceding clinically or radiographically detectable recurrence.

#### 3.3.2. Impact on Overall Survival and Disease-Free Survival

Beyond recurrence risk, multiple studies have demonstrated that mortality risk is significantly higher among patients with detectable ctDNA compared to those who are ctDNA-negative, indicating that ctDNA status is strongly associated with both overall survival (OS) and disease-free survival (DFS). In one study, post-treatment ctDNA detection was associated with significantly shorter recurrence-free survival (HR, 5.27) and OS (HR, 12.83) compared with undetectable ctDNA [23]. Similarly, in the PREDATOR study, MRD-positive patients demonstrated significantly worse DFS (HR, 4.97) and OS (HR, 27.05), further supporting the prognostic significance of persistent ctDNA following curative-intent treatment [24].

More recent studies have reinforced these findings in larger and earlier-stage CRC populations. Yang et al. reported that postoperative ctDNA positivity was associated with significantly worse DFS in patients with resectable stage I-III CRC (HR, 20.74; 95% CI, 7.19–59.83) [25]. In the large CIRCULATE-Japan GALAXY cohort, MRD positivity following surgery was similarly associated with inferior DFS (HR, 11.99) and OS (HR, 9.68) [26]. Collectively, these findings demonstrate the strong prognostic value of ctDNA for predicting both disease-free and overall survival in CRC patients.

#### 3.3.3. Role in Treatment Monitoring and Therapy Guidance

An important advantage of ctDNA monitoring in CRC patients is its ability to assess treatment response over time. Serial ctDNA sampling during adjuvant chemotherapy may reflect treatment effectiveness, as successful therapy is generally associated with a reduction or clearance of detectable ctDNA, whereas ineffective treatment may result in persistent or increasing ctDNA levels [20]. Henriksen et al. demonstrated this relationship in stage III CRC, where only patients who achieved permanent ctDNA clearance during adjuvant chemotherapy remained recurrence-free, whereas patients with transient clearance or persistent ctDNA positivity subsequently experienced recurrence [27]. Similarly, in the CIRCULATE-Japan GALAXY cohort, sustained ctDNA clearance during adjuvant chemotherapy was associated with substantially more favorable survival outcomes than transient clearance [26]. These findings support ctDNA dynamics as a biomarker for assessing therapeutic response rather than relying exclusively on a single postoperative measurement.

The potential use of ctDNA to guide treatment selection has also been investigated. Importantly, a biomarker may be strongly prognostic without being predictive of benefit from a specific therapeutic intervention. Observational evidence from the GALAXY study suggested that postoperative ctDNA-positive patients with stage II or III CRC may derive greater benefit from adjuvant chemotherapy [28]. However, observational findings may be affected by treatment-selection bias and cannot independently establish predictive utility.

Randomized trials have produced more nuanced findings. In the DYNAMIC trial, a ctDNA-guided strategy in stage II colon cancer reduced the use of adjuvant chemotherapy from 28% to 15% without compromising recurrence-free survival [29,30], supporting the feasibility of risk-adapted treatment selection. In the CIRCULATE AIO/ABCSG trial, chemotherapy did not significantly improve disease-free survival among ctDNA-positive patients in the intention-to-treat analysis, although exploratory per-protocol findings suggested possible benefit; interpretation was limited by premature trial closure and reduced statistical power [19]. In DYNAMIC-III, treatment de-escalation in ctDNA-negative stage III disease did not meet the prespecified non-inferiority criterion, while treatment escalation in ctDNA-positive patients did not improve recurrence-free survival [31]. Similarly, the phase III ALTAIR trial did not demonstrate a significant disease-free survival improvement with trifluridine/tipiracil compared with placebo in ctDNA-positive patients following standard therapy and was associated with substantially greater hematologic toxicity [18]. Collectively, these findings confirm the strong prognostic value of ctDNA but demonstrate that its predictive utility is treatment- and context-dependent and remains unproven. A positive ctDNA result should therefore not, by itself, be interpreted as a mandate for treatment modification.

ctDNA has also provided an alternative approach for assessing tumor burden and disease progression. Increased ctDNA shedding and higher circulating ctDNA levels have been associated with larger tumor size and greater tumor proliferative capacity [32]. Additional studies have similarly reported positive correlations between ctDNA levels and tumor size [33]. In several investigations, ctDNA-positive patients were found to have larger tumors than ctDNA-negative patients [34,35]. These findings suggest that ctDNA levels may reflect tumor burden and disease progression, supporting its utility as a dynamic biomarker during patient monitoring.

#### 3.3.4. Lead Time Advantage over Conventional Methods

Another advantage that ctDNA monitoring provides over traditional surveillance techniques is the lead time associated with recurrence detection. Numerous studies have demonstrated that ctDNA can identify molecular recurrence several months before conventional detection methods such as CT imaging and CEA testing, with reported mean or median lead times of approximately 2.5 to 11.5 months across CRC cohorts [15,22,27,36]. More recently, the prospective PROVENC3 study demonstrated that longitudinal ctDNA surveillance detected molecular recurrence a median of 7.6 months before clinical recurrence in patients with stage III colon cancer [37]. Given the importance of early intervention in cancer management, this lead time may provide clinicians with an opportunity to identify disease recurrence before it becomes clinically apparent. Earlier detection of recurrence may facilitate more timely clinical intervention and potentially improve patient outcomes. Consequently, the lead-time advantage provided by ctDNA monitoring represents one of the most promising clinical applications of MRD detection.

#### 3.3.5. Comparison with Established Biomarkers

With regard to traditional surveillance biomarkers such as CEA, several studies suggest that ctDNA may provide greater sensitivity for detecting MRD and recurrent disease. In a prospective study evaluating methylated *SEPTIN9* as a postoperative biomarker, Leon Arellano et al. reported that ctDNA testing performed one month after surgery demonstrated 100% sensitivity and 94.7% specificity for detecting recurrence, compared with a sensitivity of 33% and specificity of 86.4% for CEA measured six months after surgery [38]. Similar findings have been reported in rectal cancer. Lu et al. demonstrated substantially greater sensitivity for tumor-informed ctDNA-based MRD detection compared with CEA, both postoperatively (78.6% vs. 21.4%) and following adjuvant therapy (96.4% vs. 42.9%) [39]. These findings demonstrate the potential for ctDNA-based approaches to identify residual or recurrent disease that may not be detected using CEA alone.

However, ctDNA and CEA may provide complementary rather than mutually exclusive information. Wang et al. reported that postoperative methylated *SEPTIN9* ctDNA provided stronger recurrence-risk stratification than CEA alone, while combining methylated ctDNA with CEA achieved a sensitivity of 86.7% and specificity of 93.1% for 1-year recurrence prediction [40]. Mo et al. similarly observed improved prognostic performance when a six-marker methylation-based ctDNA assay was combined with CEA [41]. These findings support a multimarker approach in which ctDNA provides sensitive molecular information while CEA remains an established, inexpensive, and widely available surveillance tool. The practical value of CEA is particularly important in resource-limited healthcare settings where access to ctDNA testing, specialized laboratory infrastructure, or reimbursement may be limited. Therefore, ctDNA should currently be viewed as complementary to, rather than a universal replacement for, CEA in postoperative CRC surveillance.

#### 3.3.6. Impact of Sampling Timing and Frequency on Sensitivity

Several studies have demonstrated that the timing and frequency of ctDNA collection can significantly influence assay sensitivity and overall detection performance. Serial sampling is generally preferred over reliance on a single measurement, as longitudinal monitoring increases the opportunity to detect low levels of residual disease that may be undetectable at an individual timepoint. For example, Fan et al. reported an increase in sensitivity from 64.6% with initial assessment to 75.0% with longitudinal sampling, while specificity remained approximately 95% [42]. Similarly, a tumor-naive genomic and methylation-based assay demonstrated an increase in sensitivity from 61.1% at the landmark timepoint to 83.3% with longitudinal monitoring [16]. More recently, a methylation-based tissue-free assay demonstrated an increase in sensitivity from 58.5% at the postoperative MRD timepoint to 84.4% with longitudinal assessment [43]. Collectively, these findings demonstrate that serial ctDNA monitoring can substantially improve recurrence detection compared with a single postoperative measurement.

The timing of postoperative sampling is also an important consideration. Surgical procedures, including tumor resection, can temporarily increase levels of wild-type cfDNA as a consequence of tissue injury and cellular turnover [27]. This increase in background cfDNA may dilute the relative abundance of ctDNA and reduce assay sensitivity, particularly when blood is collected shortly after surgery [27]. Across major included studies, postoperative sampling was performed as early as 3–7 days after surgery [35,38,44], at approximately 3 weeks [11], at 1 month [38], and within 6 weeks [45]. Some studies also repeated testing at 3 months, following adjuvant therapy, or during longitudinal surveillance [38,44]. These collection schedules reflect study- and assay-specific protocols rather than a universally accepted postoperative window. Both commercial and laboratory-developed assays have been evaluated using different sampling schedules; therefore, the timing established for one platform should not be directly extrapolated to another. An appropriately timed landmark sample followed by longitudinal surveillance is preferable to reliance on a single fixed timepoint.

Biological tumor characteristics are important determinants of ctDNA shedding and detectability. Tumors with secretory features and the CMS3 metabolic subtype have been associated with lower circulating ctDNA concentrations, whereas microsatellite instability may be associated with greater ctDNA shedding [34,35]. Detection also varies according to the anatomical site of recurrence, with lower detection rates reported for pulmonary and peritoneal recurrence than for hepatic metastases. Consequently, a negative ctDNA result cannot definitively exclude MRD, particularly in patients with low-shedding tumors or recurrence at sites associated with limited ctDNA release. Negative findings should therefore be interpreted alongside tumor biology, clinicopathological risk factors, conventional surveillance, and serial ctDNA measurements rather than as proof that residual disease is absent.

### 3.4. ctDNA Detection Assay Strategies

A variety of approaches are currently available for ctDNA detection, including digital polymerase chain reaction (PCR), digital droplet PCR (ddPCR), PCR amplicon-based next-generation sequencing (NGS), hybridization capture-based NGS, epigenomic approaches, and whole-genome sequencing (WGS). Despite the growing clinical interest in ctDNA-based MRD detection, there is currently no universally accepted standard assay for clinical implementation. This lack of standardization is attributable to differences in assay sensitivity, specificity, sequencing depth, bioinformatic analysis pipelines, turnaround time, cost, and clinical validation across platforms.

PCR- and NGS-based methods formed the principal technological foundations for ctDNA analysis across the included studies. PCR-based methods, particularly ddPCR, enabled the sensitive detection and quantification of predefined low-frequency tumor-associated variants or methylated loci. In contrast, NGS-based approaches provided broader genomic coverage and enabled the simultaneous analysis of multiple mutations, methylation markers, copy-number alterations, fragmentomic features, and other tumor-derived signals. Depending on the assay design, these technologies were implemented using either tumor-informed or tumor-naive strategies.

Tumor-informed assays are developed using genomic information obtained from an individual patient’s tumor. Following tumor sequencing, patient-specific alterations are selected and subsequently tracked in plasma samples during disease monitoring. In contrast, tumor-naive assays do not require prior tumor sequencing and instead utilize predefined genomic, epigenomic, or multiomic signatures associated with CRC. Accordingly, tissue concordance should not be treated as a directly comparable performance metric across assay strategies. In tumor-informed approaches, the alterations monitored in plasma are selected from the patient’s tumor profile, making the relationship to tumor tissue inherent to assay design rather than an independent measure of performance. Tumor-naive assays are developed without prior tumor-tissue sequencing and generally do not have an equivalent tissue-concordance assessment. Because analytical platforms and assay strategies represent overlapping classifications, individual publications may be represented in more than one category. Table 2 summarizes the major ctDNA assay strategies and analytical platforms identified across the 94 included publications, together with the number of publications employing each approach, their principal methodological and clinical characteristics, and the corresponding publications. Tumor-informed/personalized assays and targeted NGS-based approaches were most frequently represented, while methylation-based, WES/WGS-enabled, and multiomic or fragmentomic strategies reflected the continued diversification of ctDNA-based MRD testing. Detailed study-level characteristics and assay performance data are provided in Supplementary Table S1.

#### 3.4.1. Tumor-Informed Assays

Tumor-informed assays are specifically designed to reflect the unique molecular profile of an individual patient’s tumor. To accomplish this, tumor tissue undergoes genomic profiling to identify patient-specific somatic mutations, which are subsequently incorporated into customized assays for longitudinal ctDNA monitoring [45,73]. Since these assays target known tumor-specific mutations, they are generally considered highly sensitive and highly specific, enabling the detection of ctDNA at extremely low frequencies [73,108]. This enhanced sensitivity improves the ability of ctDNA to predict residual disease, recurrence, and treatment response in CRC patients [73].

Despite these advantages, tumor-informed approaches have several limitations. Given that the assay is designed around a predetermined set of mutations, it is not well suited for the discovery of newly emerging mutations or significant genomic evolution occurring during disease progression [108]. Furthermore, tumor-informed assays require access to tumor tissue for initial sequencing and assay design, increasing both cost and turnaround time compared with tumor-naive approaches [6,15,73,109]. Additional practical barriers may limit tumor-informed testing in real-world settings. Archival tumor tissue may be unavailable, and formalin-fixed specimens may have low tumor cellularity, poor DNA quality, or insufficient tumor-derived DNA for reliable profiling and individualized assay development. These limitations may delay testing or prevent assay generation, particularly when rapid postoperative treatment decisions are required.

One of the most widely utilized tumor-informed assays in CRC is the Signatera assay. Signatera is a personalized, NGS-based assay that identifies patient-specific mutation signatures through tumor sequencing, often using whole-exome sequencing and targeted NGS approaches [9,32,73]. These individualized mutation panels are subsequently monitored in serial plasma samples to detect ctDNA during postoperative surveillance and MRD assessment [9]. Owing to its high sensitivity and strong clinical validation, Signatera has been widely employed in CRC studies evaluating recurrence prediction and MRD detection.

#### 3.4.2. Tumor-Naive Assays

In contrast to tumor-informed assays, tumor-naive assays do not require prior sequencing of the patient’s tumor tissue. Instead, these approaches utilize predefined panels containing genomic and epigenomic alterations that are commonly observed in CRC [73,108]. The absence of a tissue-sequencing requirement allows these assays to be implemented more rapidly and at lower cost than tumor-informed approaches [17,73]. Furthermore, tumor-naive assays may evaluate hundreds to thousands of potential alterations simultaneously, providing broader genomic coverage and facilitating implementation in situations where tumor tissue is unavailable.

Historically, tumor-naive approaches have been considered less sensitive than tumor-informed assays because they are not tailored to the specific mutation profile of an individual patient’s tumor [14,73]. However, substantial efforts have been made to improve assay performance through the incorporation of additional biomarkers, including DNA methylation signatures, fragmentomics (e.g., fragment lengths, end motifs, and nucleosome footprints), and serial ctDNA monitoring strategies [14].

Among the tumor-naive assays identified in the literature, Guardant Reveal was one of the most commonly utilized platforms for MRD detection in CRC patients [17,23,26,82,83]. Guardant Reveal combines genomic and epigenomic analyses to identify ctDNA without requiring prior tumor sequencing [80]. The assay evaluates approximately 2000 differentially methylated regions associated with CRC and integrates these findings with genomic alterations to improve detection sensitivity [17,80]. Samples are classified as either ctDNA detected (positive) or ctDNA not detected (negative) according to predefined statistical thresholds that determine whether the observed methylation and genomic patterns are likely tumor-derived [80].

Although tumor-naive approaches have traditionally been viewed as less sensitive than tumor-informed strategies, the incorporation of methylation analysis and independent genomic assessments has significantly improved their analytical performance [110]. In addition, the reported turnaround time for Guardant Reveal is approximately 7–14 days, providing a practical advantage in situations requiring rapid clinical decision-making [110].

Commercial platforms generally provide standardized workflows, centralized quality control, established reporting procedures, and broader clinical validation. However, proprietary testing costs, reliance on external laboratories, reimbursement limitations, and restricted assay customization may reduce accessibility. Laboratory-developed assays may offer greater flexibility and potentially lower direct testing costs when local infrastructure is available, but require specialized equipment, technical expertise, bioinformatic support, analytical validation, and ongoing quality assurance. Direct comparisons of performance and total cost remain difficult because economic data were inconsistently reported and assay designs, sampling schedules, and clinical settings varied substantially across studies.

**Figure 2:**
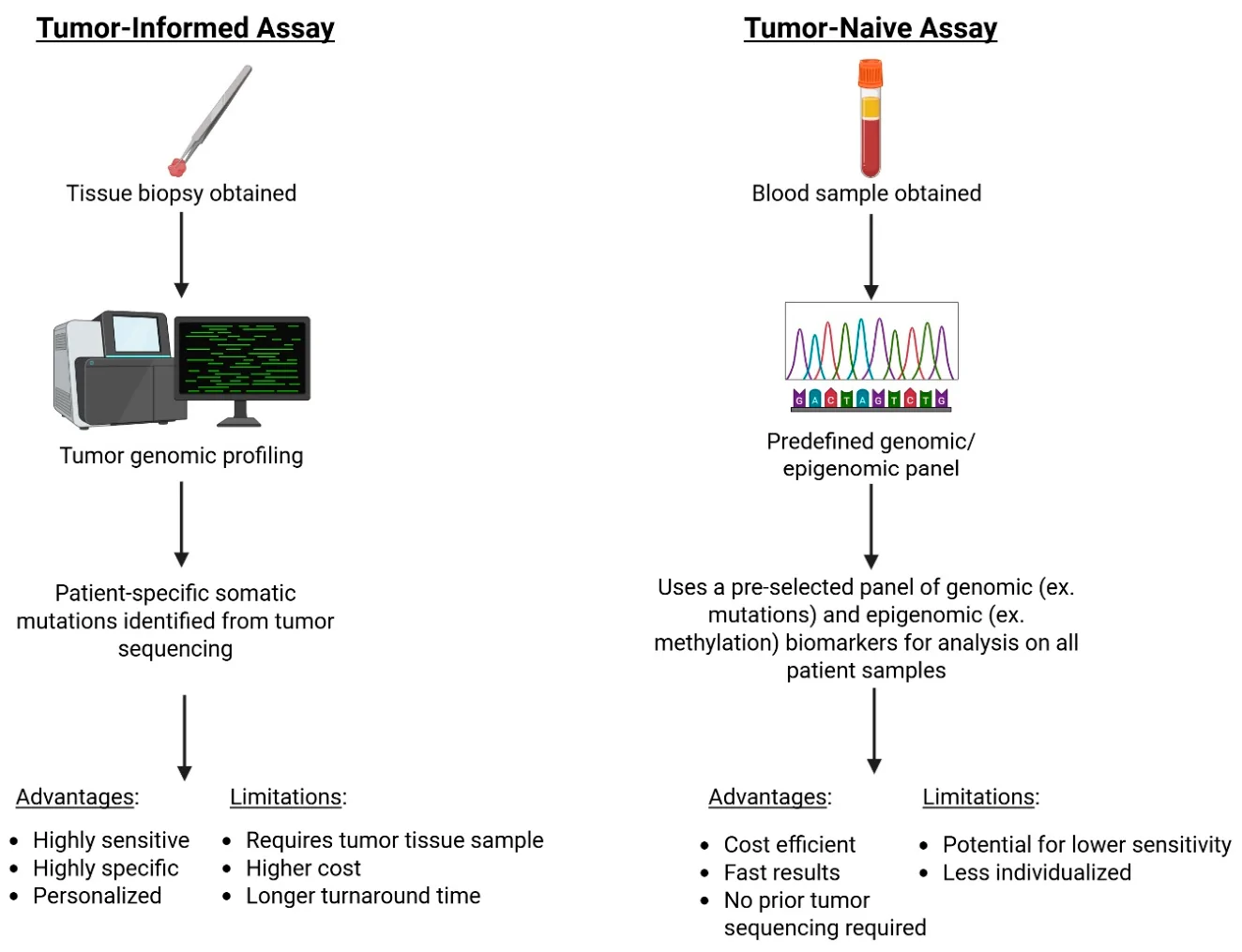
Tumor-informed versus tumor-naive ctDNA assay strategies. Tumor-informed assays utilize patient tumor tissue obtained through surgery or biopsy for genomic profiling and identification of patient-specific mutations, which are subsequently monitored in plasma samples. Tumor-naive assays do not require prior tumor sequencing and instead analyze plasma-derived ctDNA using predefined genomic and epigenomic panels containing alterations commonly associated with CRC. Potential limitations of tumor-informed assays include the requirement for adequate, high-quality tumor tissue and longer assay-development and turnaround times. Tumor-naive assays may demonstrate reduced sensitivity in certain tumor subtypes or low-shedding disease, while methylation-based approaches may generate false-positive signals arising from non-malignant methylation patterns.

Overall, both tumor-informed and tumor-naive assays possess distinct advantages and limitations, as illustrated in Figure 2. Tumor-informed approaches generally provide high analytical specificity and sensitive detection of patient-specific alterations but require tumor tissue, greater resources, and longer turnaround times. Conversely, tumor-naive assays offer faster implementation, lower costs, and greater accessibility, although their performance may vary depending on assay design, tumor biology, and the genomic or epigenomic signals incorporated. As ctDNA technologies continue to evolve, both strategies are likely to play important and complementary roles in the clinical management of CRC.

### 3.5. Variant Allele Frequency (VAF)

VAF represents the proportion of DNA fragments containing a tumor-specific mutation relative to the total DNA fragments present within a sample. One of the major challenges associated with early ctDNA detection technologies was their limited ability to reliably identify tumor-derived variants present at very low VAFs [7]. Older sequencing methods, including Sanger sequencing and pyrosequencing, generally required mutant alleles to be present at frequencies greater than 10% for reliable detection [7]. Thus, ctDNA could only be detected when tumor-derived DNA constituted a relatively large proportion of the total circulating DNA.

Advances in molecular technologies, including ddPCR, targeted NGS, ultra-deep sequencing, and error-corrected sequencing approaches, have substantially improved analytical sensitivity and enabled the detection of tumor-derived variants at increasingly low VAFs. This is particularly important for MRD applications, where ctDNA may represent only a very small fraction of total circulating cfDNA. Across the included studies, reported VAF thresholds varied substantially according to assay design. For example, thresholds or detectable VAF ranges of approximately 0.018–0.783% [73], 0.02–0.05% [55], and 0.035% [37] were reported with different ctDNA approaches, while Lu et al. reported a VAF detection threshold as low as 0.004% using a tumor-informed assay [39]. Other highly sensitive tumor-informed assays demonstrated >95% analytical sensitivity at a mean VAF of 0.01% [26]. These findings demonstrate the increasing ability of contemporary ctDNA assays to detect very low-frequency tumor-derived alterations.

VAF has also been utilized as a marker for monitoring treatment response and disease progression. Several studies have reported that variants with higher baseline VAFs may provide more robust and reproducible longitudinal measurements during serial ctDNA monitoring [11,36,111]. However, lowering the detection threshold may increase the likelihood of false-positive results arising from sequencing artifacts, background noise, or biological variation. Consequently, some investigators have prioritized tracking mutations with the highest detectable VAF within a patient’s tumor profile to improve assay specificity and reduce uncertainty in treatment monitoring. Collectively, these findings highlight the importance of balancing analytical sensitivity with specificity when selecting ctDNA targets and interpreting VAF measurements in clinical practice.

### 3.6. Specific Genomic Biomarkers in CRC

A variety of genomic biomarkers were identified across the studies included in this review and are summarized in Supplementary Table S1. Frequently evaluated genes included *APC*, *TP53*, *KRAS*, *NRAS*, *BRAF*, *PIK3CA*, *SMAD4*, *FBXW7*, *CTNNB1*, *RNF43*, *POLE*, *PTEN*, and *ERBB2* [20,22,24,25,26,36,56,58,106,111]. These genes represent recurrently altered molecular targets in CRC and were commonly incorporated into mutation-based ctDNA assays for MRD detection and longitudinal disease monitoring. Common *KRAS* variants included p.G12A, p.G12C, p.G12D, p.G12R, p.G12S, p.G12V, and p.G13D, with additional studies evaluating alterations at codon Q61 [98].

Depending on the study design, these biomarkers were detected using both tumor-informed and tumor-naive approaches. Tumor-naive assays generally utilized predefined panels containing recurrent CRC-associated alterations, whereas tumor-informed assays selected patient-specific somatic mutations identified through tumor sequencing. Consequently, the clinical utility of genomic ctDNA testing is not dependent on any single universal CRC mutation; rather, detection performance is often improved by simultaneously interrogating multiple tumor-associated alterations. This is particularly important given the molecular heterogeneity of CRC and the variability in mutation profiles between individual patients.

In addition to commonly mutated CRC driver genes, several studies tracked less frequent but clinically informative alterations, including *BRAF* V600E, *POLE* mutations, *ERBB2* amplification, *NTRK* and *RET* fusions, and *ALK* rearrangements [26,69,94,100,106]. These findings illustrate how ctDNA can provide information beyond MRD status by enabling longitudinal assessment of molecular evolution and potentially actionable tumor alterations. For example, the large GALAXY analysis included alterations such as *BRAF* V600E, *KRAS* G12C, *ERBB2* amplification, *TP53* Y220C, *NTRK1/2/3* fusions, and *RET* fusions within its molecular characterization [26].

More broadly, detectable ctDNA itself emerged as a clinically important biomarker across numerous studies. Rather than relying on the presence of a single mutation, many contemporary assays define MRD positivity according to the detection of one or more patient-specific SNVs, structural variants, or other tumor-derived molecular signals [20,69,83]. Longitudinal monitoring of these alterations enables the assessment of MRD status, recurrence risk, disease progression, and treatment response.

### 3.7. Epigenetic Biomarkers of ctDNA in CRC

DNA methylation regulates gene expression and maintains genomic stability. Dysregulation of methylation is frequently observed in cancer, driving the initiation and progression of cancer [112]. These aberrant methylation changes can also be observed in ctDNA and have been shown to reflect the tumor cells or tissues of origin [113]. The use of methylated ctDNA as a biomarker has become an emerging approach in the detection of MRD, as tumor-associated methylation patterns are often more stable throughout tumor development and are frequently observed across cancer types [113].

Several studies have evaluated targeted methylation markers for MRD detection and recurrence prediction in CRC. For example, Wang et al. utilized a methylated *SEPTIN9* (mS9) assay as a molecular indicator of postoperative MRD and relapse risk [40]. Postoperative mS9 positivity was significantly associated with reduced disease-free survival and increased recurrence risk across both early and extended postoperative periods. Prognostic performance was further improved when methylated ctDNA was combined with CEA. Similarly, Mo et al. evaluated a six-marker methylation-based ctDNA assay in patients with stage I–III CRC [41]. Methylated ctDNA was identified as a strong independent predictor of recurrence, with ctDNA-positive patients demonstrating a significantly greater recurrence risk as early as one month following surgery. Prognostic performance was again improved when methylated ctDNA was combined with CEA.

Other targeted methylation strategies have demonstrated similar potential. Benhaim et al. evaluated methylated *WIF1* and *NPY* together with patient-specific ctDNA mutations in stage II–III CRC, demonstrating the feasibility of integrating methylation and mutation signals for postoperative MRD assessment [35]. More recently, do Canto et al. developed a methylation-specific ddPCR multiplex assay that achieved a reported sensitivity of 64.4% in localized CRC and specificity of 96.7%, further supporting the potential of targeted methylation markers for sensitive ctDNA detection [87].

Beyond individual methylated loci, tissue-free epigenomic assays now evaluate large numbers of differentially methylated regions and can be combined with genomic signals to improve MRD detection. Nakamura et al. demonstrated that a tumor-naive genomic and methylation-based assay achieved a sensitivity of 61.1% at a postoperative timepoint, increasing to 83.3% with longitudinal monitoring [16]. A subsequent methylation-based tissue-free assay evaluated in the GALAXY cohort demonstrated a postoperative MRD sensitivity of 58.5%, which increased to 84.4% with longitudinal assessment, with reported longitudinal specificity of 92.1% at the patient level and 97.2% at the sample level [43].

Large tissue-free studies further support the clinical relevance of epigenomic ctDNA detection. The TRACC Part B study incorporated genomic and methylation signals within a multiomic tissue-free assay, while the phase III Alliance N0147 analysis evaluated an epigenomic Guardant Reveal assay in more than 2000 patients with stage III colon cancer [82,83]. These studies illustrate the increasing shift from single-marker methylation assays toward broader epigenomic and multiomic strategies for MRD assessment.

Despite these advantages, methylation-based assays also have important limitations. Methylation patterns are not always cancer-specific, and overlapping aberrant methylation associated with non-malignant conditions may produce false-positive results [78]. Furthermore, differences in marker selection, assay platforms, bioinformatic algorithms, and positivity thresholds currently limit direct comparison across studies. Overall, the available evidence suggests that methylation provides a valuable complementary molecular signal for ctDNA-based MRD detection and may be particularly useful in tumor-naive and multiomic approaches where mutation-only detection may have limited sensitivity.

### 3.8. Implications of ctDNA Use in CRC Clinical Practice

The findings reviewed in this study suggest that ctDNA has the potential to significantly influence the clinical management of CRC by enabling more personalized approaches to postoperative surveillance and treatment decision-making. As a sensitive biomarker of MRD, ctDNA provides clinicians with molecular evidence of residual disease that may not be detectable using conventional imaging or serum biomarkers alone.

One of the most promising applications of ctDNA is its ability to stratify patients according to recurrence risk following curative-intent treatment. Patients with detectable postoperative ctDNA consistently represent a population at substantially increased risk of recurrence and may therefore be candidates for intensified surveillance, consideration of additional therapeutic strategies, or enrollment in clinical trials. Conversely, persistently ctDNA-negative patients may represent a lower-risk population in whom treatment de-escalation strategies could potentially be explored. In this manner, ctDNA-guided management has the potential to support more individualized treatment strategies while reducing unnecessary treatment and associated toxicity.

However, the prognostic value of ctDNA should not be equated with predictive utility. Although postoperative ctDNA positivity consistently identifies patients at elevated recurrence risk, current randomized evidence from DYNAMIC, CIRCULATE, DYNAMIC-III, and ALTAIR has produced mixed findings regarding the clinical benefit of ctDNA-directed treatment escalation or de-escalation. These results indicate that the benefit of treatment modification depends on the disease setting, therapeutic intervention, and trial design. For example, observational evidence from the GALAXY study suggests that postoperative ctDNA-positive patients may derive greater benefit from adjuvant chemotherapy [28]. Randomized evidence from DYNAMIC supports the feasibility of ctDNA-guided treatment selection in stage II colon cancer, where reduced chemotherapy use did not compromise recurrence-free survival [30]. In contrast, DYNAMIC-III did not establish non-inferiority for treatment de-escalation in ctDNA-negative stage III disease, while treatment escalation in ctDNA-positive patients did not improve recurrence-free survival [31]. Similarly, the randomized phase III ALTAIR trial did not demonstrate a significant improvement in disease-free survival with trifluridine/tipiracil initiated solely on the basis of postoperative ctDNA positivity [18]. These findings indicate that further prospective randomized evidence is required to define how MRD status should be used to select, intensify, or de-escalate treatment.

Beyond risk stratification, ctDNA monitoring may facilitate dynamic assessment of treatment response through serial sampling. Changes in ctDNA levels over time may provide clinicians with valuable information regarding disease status and therapeutic effectiveness. In particular, sustained ctDNA clearance has been associated with more favorable outcomes, whereas persistent or recurrent ctDNA positivity is generally associated with greater recurrence risk [26,27]. This dynamic information may eventually enable treatment strategies to be adapted according to molecular response rather than relying solely on radiographic findings.

Despite these advantages, ctDNA-guided management has not yet been fully integrated into routine clinical practice. Important barriers include variability in assay methodology and sensitivity, differences in postoperative sampling schedules, cost and turnaround time, uncertainty regarding the optimal response to a positive ctDNA result, and the absence of standardized clinical algorithms for ctDNA-directed management. Additional prospective clinical trials, assay standardization, and consensus guidelines will therefore be required before ctDNA can be widely adopted as a routine component of CRC management. Nevertheless, the current evidence strongly supports ctDNA as a promising biomarker for advancing precision oncology and postoperative surveillance in CRC.

### 3.9. Limitations and Future Directions

Despite the promising findings supporting ctDNA as a biomarker for MRD detection in CRC, several limitations currently hinder its widespread clinical implementation. Key challenges identified throughout the literature include variability in study size and design, insufficient long-term follow-up in some cohorts, methodological heterogeneity, limited prospective evidence demonstrating the clinical benefit of ctDNA-directed treatment, and incomplete standardization of sampling and assay interpretation.

Although several early studies included relatively small patient cohorts, including individual case reports and studies involving fewer than five patients, the evidence base has increasingly expanded to include large prospective and multicenter cohorts involving hundreds or thousands of patients. For example, recent studies such as CIRCULATE-Japan GALAXY, CIRCULATE, and Alliance N0147 have evaluated ctDNA in substantially larger patient populations [19,26,82]. Nevertheless, many individual assay platforms and specific clinical applications remain supported by relatively small or selected cohorts, limiting subgroup analyses and generalizability. Continued validation in large, prospectively enrolled and independent patient populations will therefore remain important.

Another recurring limitation is variability in follow-up duration and sampling schedules. Some studies monitored patients for relatively short periods following ctDNA assessment, potentially underestimating later recurrence events. Even among studies with extended follow-up, differences in the timing and frequency of postoperative blood collection may substantially influence ctDNA detection and reported assay performance. As demonstrated throughout this review, longitudinal sampling frequently improves sensitivity compared with single landmark measurements. Future studies should therefore establish standardized postoperative sampling windows and longitudinal surveillance schedules to facilitate more meaningful comparison across trials.

Methodological heterogeneity remains one of the most significant barriers to cross-study comparison and clinical standardization. Differences in assay design, sequencing depth, DNA input, number and type of biomarkers analyzed, bioinformatic pipelines, positivity thresholds, and reporting practices complicate direct comparisons between studies. This heterogeneity is reflected in the wide range of reported clinical sensitivities and specificities observed across the literature. Future efforts should focus on developing consensus standards for assay validation, analytical reporting, sampling procedures, and interpretation of MRD positivity.

Direct comparison with established surveillance approaches also remains important. Although several studies now demonstrate that ctDNA can outperform or complement CEA and can detect recurrence months before conventional imaging, head-to-head comparisons remain heterogeneous with respect to assay type, testing interval, clinical endpoint, and surveillance strategy. Future studies should evaluate ctDNA prospectively alongside CEA, CT, MRI, and other established surveillance modalities to determine whether ctDNA should supplement, modify, or partially replace components of current postoperative surveillance.

Although the included studies represent multiple geographic regions, demographic and healthcare-system representation remains uneven. Many investigations were conducted at specialized academic centers or within well-resourced healthcare systems. Broader validation across diverse populations, community-based clinical settings, and healthcare systems with different levels of access to molecular testing will be necessary to establish the generalizability, accessibility, and cost-effectiveness of ctDNA-guided surveillance.

Finally, continued technological development will be important for improving the sensitivity and accessibility of ctDNA testing (Figure 2). Tumor-informed assays offer sensitive detection of patient-specific molecular alterations but require tumor tissue and individualized assay development. Tumor-naive approaches avoid these requirements and may offer faster and more scalable testing, although performance remains dependent on assay design and tumor biology. The increasing incorporation of methylation signatures, fragmentomic features, structural variants, copy-number alterations, and other multiomic signals may improve detection in low-shedding disease and reduce reliance on any single class of molecular alteration. However, these emerging approaches require further prospective validation and standardization before widespread clinical implementation. As these challenges are addressed, ctDNA-based MRD detection has the potential to become an increasingly important component of precision oncology and postoperative surveillance in colorectal cancer.

## 4. Conclusions

This review critically evaluated the current evidence supporting the use of ctDNA for MRD detection and postoperative surveillance in CRC. Across the included literature, postoperative ctDNA positivity was consistently associated with increased recurrence risk, reduced disease-free survival, and poorer overall survival. Serial ctDNA monitoring also provided clinically relevant information regarding treatment response and frequently detected molecular recurrence months before conventional surveillance approaches such as imaging and CEA testing. Collectively, these findings establish ctDNA as a highly promising biomarker for recurrence prediction, prognostic assessment, treatment monitoring, and postoperative risk stratification in CRC.

A diverse range of ctDNA detection platforms, biomarkers, and assay strategies have been developed, including tumor-informed and tumor-naive approaches utilizing PCR-, NGS-, WES-, WGS-, methylation-, and multiomic-based technologies. Tumor-informed assays enable the sensitive detection of patient-specific molecular alterations, whereas tumor-naive approaches offer advantages in scalability, turnaround time, and independence from tumor tissue. The increasing incorporation of methylation signatures, fragmentomics, structural variants, and other complementary molecular signals may further improve ctDNA detection, particularly in settings characterized by low tumor shedding. However, substantial methodological heterogeneity remains, and no universally accepted standard currently exists for ctDNA-based MRD detection in CRC.

Importantly, the strong prognostic value of ctDNA does not yet establish that treatment modification based solely on ctDNA status improves clinical outcomes. Although observational studies support the potential value of ctDNA-guided treatment selection, randomized evidence remains limited and has produced mixed findings. Consequently, additional prospective randomized trials, standardized sampling schedules and assay methodologies, and consensus clinical guidelines are required to determine how ctDNA results should be incorporated into treatment escalation, de-escalation, and surveillance strategies. As these challenges are addressed, ctDNA-based MRD detection has the potential to become an increasingly important component of personalized CRC management and precision oncology.

## Figures and Tables

**Table 1:** Clinical assay performance, prognostic value, clinical utility, and analytical or operational characteristics used to evaluate ctDNA assays for MRD detection in CRC. This table summarizes commonly reported variables in ctDNA-based MRD studies and distinguishes technical assay characteristics, clinical performance, prognostic associations, and clinical-utility endpoints. Clinical performance measures describe test accuracy at a defined assessment timepoint. Prognostic measures describe associations between ctDNA status or dynamics and subsequent patient outcomes. Clinical-utility endpoints evaluate whether ctDNA-guided management changes treatment use or clinical outcomes. These domains should be interpreted separately.

Performance Domain	Variable	Definition	Reported Findings/Range and Context	Notes
Clinical performance	Sensitivity	Ability to detect MRD when it is truly present	Highly variable (~25–100%) across reported clinical settings	Influenced by assay design, sampling timepoint, disease stage, tumor shedding, and endpoint; longitudinal sampling generally improves sensitivity
Clinical performance	Specificity	Ability to rule out MRD when it is truly absent	Generally high, but variable (~63–100%)	Often >90%, although lower values were reported for some assays/settings
Clinical performance	False Positive Rate (FPR)	% of positive ctDNA results in patients without MRD	Infrequently reported directly	Low FPR is particularly important when ctDNA results are used to guide treatment
Clinical performance	False Negative Rate (FNR)	% of patients who have MRD but display negative ctDNA results	Infrequently reported directly	Influenced by low tumor shedding, sampling timing, DNA input, assay sensitivity, and metastatic site
Clinical performance	Positive Predictive Value (PPV)	Likelihood that a positive result reflects true MRD	Variable; not consistently reported	Strongly influenced by underlying recurrence prevalence and clinical setting
Clinical performance	Negative Predictive Value (NPV)	Likelihood that a negative result reflects absence of MRD	Variable; not consistently reported	A negative result does not completely exclude MRD, particularly after a single postoperative sample
Clinical performance	Lead Time	Time between ctDNA positivity and radiological/clinical recurrence	Approximately 2.5–11.5 months	ctDNA can precede recurrence detected by conventional surveillance
Analytical or operational characteristics	Variant Allele Frequency (VAF)	Proportion of cfDNA fragments containing tumor-specific alterations	Highly assay-dependent; very low VAFs (<0.1%) can be detected by some platforms	Lower detection thresholds are particularly important for MRD
Analytical or operational characteristics	Turnaround Time	Time from sample collection to result	Assay-dependent	Tumor-naive approaches generally offer faster testing; tumor-informed assays require tumor profiling and assay development
Analytical or operational characteristics	Tumor-tissue requirement/relationship to tumor profiling	Whether prior tumor-tissue sequencing is required to select alterations for plasma monitoring	Strategy-dependent; not directly comparable across tumor-informed and tumor-naive assays	Tumor-informed assays use patient-specific alterations identified through tumor profiling; therefore, their relationship to tumor tissue is inherent to assay design rather than an independent measure of performance; tumor-naive assays do not require prior tumor sequencing and generally lack an equivalent tissue-concordance assessment
Prognostic value	RFS/DFS hazard ratio	Relative hazard of recurrence, disease progression, or death according to ctDNA status	Post-treatment ctDNA positivity was consistently associated with poorer RFS or DFS; selected reported HRs were approximately 4.97–20.74	Report the endpoint, assessment timepoint, comparator, adjustment status, and 95% CI; HRs are prognostic effect estimates, not measures of assay accuracy
Prognostic value	OS hazard ratio	Relative hazard of death according to ctDNA status	Post-treatment ctDNA positivity was associated with poorer OS; selected reported HRs were approximately 9.68–27.05	Estimates vary by disease stage, treatment context, follow-up, and covariate adjustment
Prognostic value	Recurrence rate/recurrence-free interval	Proportion of patients experiencing recurrence, or remaining recurrence-free, according to ctDNA status	Recurrence was consistently more frequent among ctDNA-positive patients; examples included 79% versus 9.8%, 81% versus 46%, and a 3-year recurrence-free interval of 30% versus 77%	Recurrence proportions should be interpreted with the assessment timepoint and duration of follow-up
Prognostic value	ctDNA clearance or persistence	Change from detectable to undetectable ctDNA, or continued/recurrent detection during or after treatment	Sustained or permanent clearance was generally associated with more favorable outcomes, whereas transient clearance, persistent positivity, or renewed positivity was associated with recurrence	Requires serial sampling; definitions of clearance and sampling schedules vary across studies
Clinical utility	ctDNA-guided treatment endpoint	Effect of using ctDNA results to guide treatment selection, escalation, or de-escalation	Outcomes included chemotherapy use, treatment escalation or de-escalation, non-inferiority of recurrence-free survival, and benefit from a specified intervention; in DYNAMIC, chemotherapy use decreased from 28% to 15% without compromising recurrence-free survival; other randomized trials produced mixed or negative results	Clinical utility is distinct from prognostic value; a prognostic association does not establish that treatment modification improves outcomes

**Note:** Analytical characteristics, clinical assay performance, prognostic associations, and clinical utility are conceptually distinct. Sensitivity and specificity describe clinical test performance at a defined timepoint. Hazard ratios, recurrence rates, and ctDNA dynamics describe associations with patient outcomes and should not be interpreted as measures of assay accuracy. Clinical-utility endpoints assess whether ctDNA-guided management changes treatment use or outcomes and require prospective validation.

**Table 2:** Summary of ctDNA assay strategies and analytical platforms used for MRD detection in CRC.

Assay Strategy/Analytical Platform	No. of Publications	Synthesis of Principal Findings	Citation
Tumor-informed/personalized assays	51	Patient-specific SNVs, structural variants, or other tumor-derived alterations were selected from tumor profiling and tracked in plasma, commonly using Signatera, bespoke mPCR-NGS, Safe-SeqS, personalized PCR/ddPCR, or related platforms. These approaches generally offered high specificity and low-VAF detection, and longitudinal sampling improved recurrence detection. Key limitations were dependence on tumor tissue, assay-development time, cost, and the possibility of missing newly emerging untracked alterations.	[9,11,12,13,14,15,18,19,20,22,24,25,26,27,28,32,34,35,37,39,42,46,47,48,49,50,51,52,53,54,55,56,57,58,59,60,61,62,63,64,65,66,67,68,69,70,71,72,73,74,75]
Tumor-naive/tissue-free assays	20	Predefined mutation, epigenomic, or fixed-panel assays avoided prior tumor sequencing and supported faster, more scalable testing. Performance was more variable than for personalized assays, particularly at a single postoperative landmark time point, but sensitivity generally improved with serial sampling and the addition of methylation or other signals. Specificity was often high, although results varied across platforms and tumor sites.	[14,17,23,25,26,33,42,43,56,58,76,77,78,79,80,81,82,83,84,85]
Methylation/ epigenomic assays	17	Studies evaluated *SEPTIN9*/mS9, WIF1/NPY, multi-marker methylation panels, differentially methylated regions, and tissue-free epigenomic signatures. Methylated ctDNA was repeatedly associated with recurrence risk and poorer disease-free outcomes. Performance improved in several studies when methylation was combined with genomic signals, serial sampling, or CEA, although overlapping methylation from non-cancer states may produce false-positive results.	[17,23,34,35,38,40,41,43,77,80,81,82,83,84,86,87,88]
Multiomic/ fragmentomic/CNA assays	10	These assays integrated genomic variants with methylation, copy-number alterations, fragmentomic features, or multiple sequencing signals. The principal rationale was to improve sensitivity in tissue-free settings and in low-shedding disease while preserving specificity. The approaches remain heterogeneous in analytes, bioinformatic pipelines, thresholds, and clinical endpoints, and require further prospective validation.	[17,23,26,58,80,81,82,83,84,89]
PCR/ddPCR/ dPCR platforms	25	Targeted PCR-based approaches provided rapid and comparatively economical detection or quantification of known mutations, structural variants, or methylated loci at low allele fractions. Specificity was frequently high, but sensitivity varied with target selection, tumor shedding, DNA input, sampling time, and whether serial samples were available. Limited marker breadth can reduce detection in genetically heterogeneous tumors.	[15,21,34,35,45,60,68,72,76,86,87,90,91,92,93,94,95,96,97,98,99,100,101,102,103]
Targeted NGS/mPCR-NGS/deep sequencing	42	Multiplex sequencing enabled simultaneous tracking of multiple variants and broader genomic coverage and was used in both tumor-informed and fixed-panel strategies. Performance depended on panel size, sequencing depth, molecular error suppression, bioinformatic filtering, and sampling frequency. Methodological heterogeneity across panels and reporting practices limits direct cross-study comparison.	[12,14,17,18,19,20,22,26,27,28,32,33,34,42,44,46,47,49,51,55,57,60,62,64,67,70,75,77,78,80,83,84,89,92,93,96,100,102,104,105,106,107]
WES/WGS-enabled assays	23	WES or WGS was used to design personalized assays, identify somatic or structural-variant targets, support whole-genome error-corrected detection, and develop tumor-agnostic approaches. These methods expanded the number and diversity of trackable alterations and supported very-low-VAF detection, but generally required greater computational resources, cost, DNA input, and turnaround time.	[11,12,15,25,27,28,37,39,45,46,47,51,53,54,59,60,62,68,71,72,74,79,89]

Note: Categories are overlapping and not mutually exclusive. Individual publications may be represented in more than one row when they employ multiple assay strategies or analytical platforms. Therefore, row counts and citation lists should not be summed, and their combined total may exceed the 94 publications included in the review.

## Data Availability

No new data were created or analyzed in this study. Data sharing is not applicable to this article.

## Supplementary Materials

The following supporting information can be downloaded at: https://www.mdpi.com/article/10.3390/curroncol33100606/s1, Table S1: Characteristics, study design, clinical assessment context, circulating tumor DNA methodologies, biomarkers, and reported performance of studies evaluating minimal residual disease in colorectal cancer [9,11,12,13,14,15,16,17,18,19,20,21,22,23,24,25,26,27,28,29,30,31,32,33,34,35,36,37,38,39,40,41,42,43,44,45,46,47,48,49,50,51,52,53,54,55,56,57,58,59,60,61,62,63,64,65,66,67,68,69,70,71,72,73,74,75,76,77,78,79,80,82,83,84,85,86,87,88,89,90,91,92,93,94,95,96,97,98,100,101,102,103,104,105,106,107,111].

## References

[1] Bray F., Laversanne M., Sung H., Ferlay J., Siegel R.L., Soerjomataram I., Jemal A. (2024). Global cancer statistics 2022: GLOBOCAN estimates of incidence and mortality worldwide for 36 cancers in 185 countries. CA Cancer J. Clin..

[2] Piña-Sánchez P., Chávez-González A., Ruiz-Tachiquín M., Vadillo E., Monroy-García A., Montesinos J.J., Grajales R., Gutiérrez de la Barrera M., Mayani H. (2021). Cancer Biology, Epidemiology, and Treatment in the 21st Century: Current Status and Future Challenges From a Biomedical Perspective. Cancer Control.

[3] Olenius T., Koskenvuo L., Koskensalo S., Lepistö A., Böckelman C. (2022). Long-term survival among colorectal cancer patients in Finland, 1991–2015: A nationwide population-based registry study. BMC Cancer.

[4] Ellison L.F., Saint-Jacques N. (2023). Five-year cancer survival by stage at diagnosis in Canada. Health Rep..

[5] Kasi P.M., Sawyer S., Guilford J., Munro M., Ellers S., Wulff J., Hook N., Krinshpun S., Malashevich A.K., Malhotra M. (2021). BESPOKE study protocol: A multicentre, prospective observational study to evaluate the impact of circulating tumour DNA guided therapy on patients with colorectal cancer. BMJ Open.

[6] Pu W., Chen F., Tang Y., Qu Y., Han Y., Zha J., Jin J., Kong F. (2024). Potential value of detection of minimal residual disease in colorectal cancer following radical resection. Chin. J. Cancer Res..

[7] Jácome A.A., Johnson B. (2023). Minimal Residual Disease in Colorectal Cancer: Are We Finding the Needle in a Haystack?. Cells.

[8] Ignatiadis M., Reinholz M. (2011). Minimal residual disease and circulating tumor cells in breast cancer. Breast Cancer Res..

[9] Eslinger C., Wu C., Ahn D.H. (2024). Minimal residual disease monitoring via ctDNA: A case report of Lynch syndrome with synchronous colorectal cancer and review of literature. J. Gastrointest. Oncol..

[10] Schraa S.J., van Rooijen K.L., van der Kruijssen D.E.W., Rubio Alarcón C., Phallen J., Sausen M., Simmons J., Coupé V.M.H., van Grevenstein W.M.U., Elias S. (2020). Circulating tumor DNA guided adjuvant chemotherapy in stage II colon cancer (MEDOCC-CrEATE): Study protocol for a trial within a cohort study. BMC Cancer.

[11] Ryoo S.-B., Heo S., Lim Y., Lee W., Cho S.H., Ahn J., Kang J.-K., Kim S.Y., Kim H.-P., Bang D. (2023). Personalised circulating tumour DNA assay with large-scale mutation coverage for sensitive minimal residual disease detection in colorectal cancer. Br. J. Cancer.

[12] Qi Z., Li Y., Wang Z., Tan X., Zhou Y., Li Z., Zhao W., Zheng X., Yao J., Li F. (2023). Monitoring of gastrointestinal carcinoma via molecular residual disease with circulating tumor DNA using a tumor-informed assay. Cancer Med..

[13] Nguyen H.T., Nguyen T.V., Nguyen Hoang V.-A., Tran D.H., Le Trinh N.A., Le M.T., Nguyen Tran T.-A., Pham T.H., Dinh T.C., Nguyen T.S. (2022). Tumor genomic profiling and personalized tracking of circulating tumor DNA in Vietnamese colorectal cancer patients. Front. Oncol..

[14] Jiang H., Huang F., Yang Y., Chen X., Shen M., Zhang C., Pan B., Wang B., Guo W. (2023). Postoperative circulating tumor DNA testing based on tumor naïve strategy after liver metastasis surgery in colorectal cancer patients. Front. Oncol..

[15] Schøler L.V., Reinert T., Ørntoft M.-B.W., Kassentoft C.G., Árnadóttir S.S., Vang S., Nordentoft I., Knudsen M., Lamy P., Andreasen D. (2017). Clinical Implications of Monitoring Circulating Tumor DNA in Patients with Colorectal Cancer. Clin. Cancer Res..

[16] Nakamura Y., Kaneva K., Lo C., Neems D., Freaney J.E., Boulos H., Hyun S.W., Islam F., Yamada-Hanff J., Driessen T.M. (2025). A Tumor-Naïve ctDNA Assay Detects Minimal Residual Disease in Resected Stage II or III Colorectal Cancer and Predicts Recurrence: Subset Analysis from the GALAXY Study in CIRCULATE-Japan. Clin. Cancer Res..

[17] Jain S., Dawood S., Lavingia V., Aderka D., Tahover E., Hsieh Y.-Y., Temper M., Goldman A., Akasheh M.A., Olsen S. (2024). Utilization of tissue-free minimal residual disease testing in colorectal cancer patients from Asia and Middle East. Front. Oncol..

[18] Bando H., Watanabe J., Takahashi Y., Kotaka M., Matsuhashi N., Oki E., Komatsu Y., Shiozawa M., Hirata K., Miyamoto Y. (2026). Post-adjuvant chemotherapy in ctDNA-positive patients with resected colorectal cancer: A randomized phase 3 trial. Nat. Med..

[19] Folprecht G., Stasik S., Reinacher-Schick A., Weiss L., Goekkurt E., Jacobasch L., Conradi L., Kröcher A., Hofheinz R.-D., Liersch R. (2026). Chemotherapy for patients with circulating tumour DNA-positive, stage II colon cancer (CIRCULATE)—An AIO/ABCSG trial. Ann. Oncol..

[20] Tie J., Cohen J.D., Wang Y., Christie M., Simons K., Lee M., Wong R., Kosmider S., Ananda S., McKendrick J. (2019). Circulating Tumor DNA Analyses as Markers of Recurrence Risk and Benefit of Adjuvant Therapy for Stage III Colon Cancer. JAMA Oncol..

[21] Kalil J.A., Krzywon L., Petrillo S.K., Tsamchoe M., Zlotnik O., Lazaris A., Metrakos P. (2024). Feasibility of ctDNA in detecting minimal residual disease and predicting recurrence for colorectal cancer liver metastases. Front. Oncol..

[22] Tie J., Wang Y., Tomasetti C., Li L., Springer S., Kinde I., Silliman N., Tacey M., Wong H.-L., Christie M. (2016). Circulating tumor DNA analysis detects minimal residual disease and predicts recurrence in patients with stage II colon cancer. Sci. Transl. Med..

[23] Parikh A.R., Chee B.H., Tsai J., Rich T.A., Price K.S., Patel S.A., Zhang L., Ibrahim F., Esquivel M., Van Seventer E.E. (2024). Minimal Residual Disease using a Plasma-Only Circulating Tumor DNA Assay to Predict Recurrence of Metastatic Colorectal Cancer Following Curative Intent Treatment. Clin. Cancer Res..

[24] Lonardi S., Nimeiri H., Xu C., Zollinger D.R., Madison R.W., Fine A.D., Gjoerup O., Rasola C., Angerilli V., Sharma S. (2022). Comprehensive Genomic Profiling (CGP)-Informed Personalized Molecular Residual Disease (MRD) Detection: An Exploratory Analysis from the PREDATOR Study of Metastatic Colorectal Cancer (mCRC) Patients Undergoing Surgical Resection. Int. J. Mol. Sci..

[25] Yang J., Yu C., Li H., Peng D., Zhou Q., Yao J., Lv J., Fang S., Shi J., Wei Y. (2024). Evaluation of Molecular Residual Disease by a Fixed Panel in Resectable Colorectal Cancer. Cancer Res. Treat..

[26] Nakamura Y., Watanabe J., Akazawa N., Hirata K., Kataoka K., Yokota M., Kato K., Kotaka M., Kagawa Y., Yeh K.-H. (2024). ctDNA-based molecular residual disease and survival in resectable colorectal cancer. Nat. Med..

[27] Henriksen T.V., Tarazona N., Frydendahl A., Reinert T., Gimeno-Valiente F., Carbonell-Asins J.A., Sharma S., Renner D., Hafez D., Roda D. (2022). Circulating Tumor DNA in Stage III Colorectal Cancer, beyond Minimal Residual Disease Detection, toward Assessment of Adjuvant Therapy Efficacy and Clinical Behavior of Recurrences. Clin. Cancer Res..

[28] Kotani D., Oki E., Nakamura Y., Yukami H., Mishima S., Bando H., Shirasu H., Yamazaki K., Watanabe J., Kotaka M. (2023). Molecular residual disease and efficacy of adjuvant chemotherapy in patients with colorectal cancer. Nat. Med..

[29] Tie J., Cohen J.D., Lahouel K., Lo S.N., Wang Y., Kosmider S., Wong R., Shapiro J., Lee M., Harris S. (2022). Circulating Tumor DNA Analysis Guiding Adjuvant Therapy in Stage II Colon Cancer. N. Engl. J. Med..

[30] Tie J., Wang Y., Lo S.N., Lahouel K., Cohen J.D., Wong R., Shapiro J.D., Harris S.J., Khattak A., Burge M.E. (2025). Circulating tumor DNA analysis guiding adjuvant therapy in stage II colon cancer: 5-year outcomes of the randomized DYNAMIC trial. Nat. Med..

[31] Tie J., Wang Y., Loree J.M., Cohen J.D., Wong R., Price T., Tebbutt N.C., Gebski V., Espinoza D., Burge M. (2025). Circulating tumor DNA-guided adjuvant therapy in locally advanced colon cancer: The randomized phase 2/3 DYNAMIC-III trial. Nat. Med..

[32] Kataoka K., Mori K., Nakamura Y., Watanabe J., Akazawa N., Hirata K., Yokota M., Kato K., Kotaka M., Yamazaki K. (2024). Survival benefit of adjuvant chemotherapy based on molecular residual disease detection in resected colorectal liver metastases: Subgroup analysis from CIRCULATE-Japan GALAXY. Ann. Oncol..

[33] Pellini B., Pejovic N., Feng W., Earland N., Harris P.K., Usmani A., Szymanski J.J., Qaium F., Mudd J., Petty M. (2021). ctDNA MRD Detection and Personalized Oncogenomic Analysis in Oligometastatic Colorectal Cancer From Plasma and Urine. JCO Precis. Oncol..

[34] Andersen L., Kisistók J., Henriksen T.V., Bramsen J.B., Reinert T., Øgaard N., Mattesen T.B., Birkbak N.J., Andersen C.L. (2024). Exploring the biology of ctDNA release in colorectal cancer. Eur. J. Cancer.

[35] Benhaim L., Bouché O., Normand C., Didelot A., Mulot C., Corre D.L., Garrigou S., Djadi-Prat J., Wang-Renault S.-F., Perez-Toralla K. (2021). Circulating tumor DNA is a prognostic marker of tumor recurrence in stage II and III colorectal cancer: Multicentric, prospective cohort study (ALGECOLS). Eur. J. Cancer.

[36] Reinert T., Petersen L.M.S., Henriksen T.V., Larsen M.Ø., Rasmussen M.H., Johansen A.F.B., Øgaard N., Knudsen M., Nordentoft I., Vang S. (2022). Circulating tumor DNA for prognosis assessment and postoperative management after curative-intent resection of colorectal liver metastases. Int. J. Cancer.

[37] Rubio-Alarcón C., Georgiadis A., Franken I.A., Wang H., van Nassau S.C.M.W., Schraa S.J., van der Kruijssen D.E.W., van Rooijen K., Linders T.C., Delis-van Diemen P. (2026). Circulating tumour DNA in patients with stage III colon cancer: Multicentre prospective PROVENC3 study. BJS.

[38] Leon Arellano M., García-Arranz M., Guadalajara H., Olivera-Salazar R., Valdes-Sanchez T., García-Olmo D. (2022). Analysis of Septin 9 Gene Hypermethylation as Follow-Up Biomarker of Colorectal Cancer Patients after Curative Surgery. Diagnostics.

[39] Lu Y., Chen J., Chen G., Peng H., Zhang J. (2026). Real-World Experience of Circulating Tumor DNA Testing in Extended Pelvic Exenteration: A Chinese Single-Institution Observational Study. J. Surg. Oncol..

[40] Wang P., Peng L., Hu X., Liu Y., Jiang T., Yin W., Wu S., Lin J. (2026). Early prediction of minimal residual disease in colorectal cancer via SEPTIN9 methylation status in circulating tumor DNA. Ann. Med..

[41] Mo S., Ye L., Wang D., Han L., Zhou S., Wang H., Dai W., Wang Y., Luo W., Wang R. (2023). Early Detection of Molecular Residual Disease and Risk Stratification for Stage I to III Colorectal Cancer via Circulating Tumor DNA Methylation. JAMA Oncol..

[42] Fan W., Xia Z., Chen R., Lin D., Li F., Zheng Y., Luo J., Xiong Y., Yu P., Gao W. (2024). Circulating tumor DNA analysis predicts recurrence and avoids unnecessary adjuvant chemotherapy in I–IV colorectal cancer. Ther. Adv. Med. Oncol..

[43] Nakamura Y., Reiter J.G., Natarajan P., Babiarz J., Srinivasan P., Joshi J., Lin Y., Chen T.-C., Liang N., Maw S. (2026). Validation of a methylation-based, tissue-free MRD assay in colorectal cancer patients from the GALAXY study. npj Precis. Oncol..

[44] Chen G., Peng J., Xiao Q., Wu H.-X., Wu X., Wang F., Li L., Ding P., Zhao Q., Li Y. (2021). Postoperative circulating tumor DNA as markers of recurrence risk in stages II to III colorectal cancer. J. Hematol. Oncol..

[45] Li X., Liu T., Bacchiocchi A., Li M., Cheng W., Wittkop T., Mendez F.L., Wang Y., Tang P., Yao Q. (2024). Ultra-sensitive molecular residual disease detection through whole genome sequencing with single-read error correction. EMBO Mol. Med..

[46] Alden S.L., Dhani H., Palsuledesai C.C., Krinshpun S., Jurdi A., Christenson E., Browner I., Rosner S. (2022). Post-operative ctDNA monitoring in stage I colon cancer: A case report. Front. Oncol..

[47] Ando K., Hamabe A., Nakamura Y., Watanabe J., Hirata K., Kataoka K., Miyo M., Kato K., Akazawa N., Kagawa Y. (2026). Molecular Residual Disease and Recurrence in Rectal Cancer Patients Undergoing Upfront Surgery. Ann. Surg..

[48] Azzi G., Slavin T.P., Izaguirre-Carbonell J., Sloane H.S., Edelstein D.L., Ma C.X. (2025). Advanced Minimal Residual Disease Detection Using a Novel Circulating Tumor DNA Assay: A Report of Two Cases. Case Rep. Oncol..

[49] Bikhchandani M., Amersi F., Hendifar A., Gangi A., Osipov A., Zaghiyan K., Atkins K., Cho M., Aguirre F., Hazelett D. (2023). POLE-Mutant Colon Cancer Treated with PD-1 Blockade Showing Clearance of Circulating Tumor DNA and Prolonged Disease-Free Interval. Genes.

[50] Burley N., Lee Y., Liu L., Gangi A., Nasseri Y., Atkins K., Zaghiyan K., Murrell Z., Osipov A., Hendifar A. (2024). ctDNA-guided adjuvant immunotherapy in colorectal cancer. Immunotherapy.

[51] Chakrabarti S., Cohen S.A., Tin A., Dangl A., Chung K.Y., Tejani M.A., Fakih M.G., Chandana S.R., Donahue C.A., George V. (2026). Utility of Circulating Tumor DNA to Assess Tumor Response in Patients with Locally Advanced Rectal Cancer Undergoing Neoadjuvant Therapy. Cancers.

[52] Emiloju O.E., Storandt M., Zemla T., Tran N., Jethwa K., Mahipal A., Mitchell J., Thiels C., Mathis K., McWilliams R. (2024). Tumor-Informed Circulating Tumor DNA for Minimal Residual Disease Detection in the Management of Colorectal Cancer. JCO Precis. Oncol..

[53] Franken I.A., Heilijgers F., Bakker M.-C.E., Rubio-Alarcón C., der Baan F.H.v., Lemmens M., Diemen P.D., Sausen M., Meijer G.A., Koopman M. (2026). Added value of tumor–stroma ratio to postsurgery circulating tumor DNA and pTN stage in risk stratification of patients with stage III colon cancer treated with adjuvant chemotherapy. ESMO Open.

[54] Frydendahl A., Widman A.J., Øgaard N., Arora A., Halmos D., Nors J., Ahrenfeldt J., Henriksen T.V., Demuth C., Raaby L. (2025). Whole-genome sequencing of cell-free DNA reveals DNA of tumor origin in plasma from patients with colorectal adenomas. Mol. Oncol..

[55] Gao Z., Huang D., Chen H., Yang Y., An K., Ding C., Yuan Z., Zhai Z., Niu P., Gao Q. (2023). Development and validation of postoperative circulating tumor DNA combined with clinicopathological risk factors for recurrence prediction in patients with stage I-III colorectal cancer. J. Transl. Med..

[56] Hisamatsu Y., Ando K., Kudo K., Nakanishi R., Kawazoe T., Zaitsu Y., Kusumoto T., Hata T., Kagawa Y., Yoshizumi T. (2025). A 14-Gene Panel for Predicting Colorectal Cancer Recurrence Using Circulating Tumor DNA in Different Testing Conditions. Cancer Sci..

[57] Kim J.K., Alden A.J., Knaus S., Thakkar R., Moudgill L., Chudzinski A., Cavallaro P., Martinez C., Bennett R.D., Marcet J. (2025). Circulating Tumor DNA Detects Minimal Residual Disease in Patients with Locally Advanced Rectal Cancer After Total Neoadjuvant Therapy. Cancers.

[58] Liu W., Li Y., Tang Y., Song Q., Wang J., Li N., Chen S., Shi J., Wang S., Li Y. (2022). Response prediction and risk stratification of patients with rectal cancer after neoadjuvant therapy through an analysis of circulating tumour DNA. eBioMedicine.

[59] Loeffler C.M.L., Bando H., Sainath S., Muti H.S., Jiang X., van Treeck M., Reitsam N.G., Carrero Z.I., Meneghetti A.R., Nishikawa T. (2025). HIBRID: Histology-based risk-stratification with deep learning and ctDNA in colorectal cancer. Nat. Commun..

[60] Loupakis F., Sharma S., Derouazi M., Murgioni S., Biason P., Rizzato M.D., Rasola C., Renner D., Shchegrova S., Koyen Malashevich A. (2021). Detection of Molecular Residual Disease Using Personalized Circulating Tumor DNA Assay in Patients With Colorectal Cancer Undergoing Resection of Metastases. JCO Precis. Oncol..

[61] Lutfi A., Afghan M.K., Kasi P.M. (2023). Circulating Tumor DNA Response and Minimal Residual Disease Assessment in DNA Polymerase Epsilon-Mutated Colorectal Cancer Undergoing Immunotherapy. Cureus.

[62] Molinari C., Marisi G., Laliotis G., Spickard E., Rapposelli I.G., Petracci E., George G.V., Dutta P., Sharma S., Malhotra M. (2024). Assessment of circulating tumor DNA in patients with locally advanced rectal cancer treated with neoadjuvant therapy. Sci. Rep..

[63] Nguyen H.T., Hoang V.-A.N., Nguyen T.V., Trinh N.A.L., Pham T.H., Le D.N., Ho H.H., Nguyen T.D., Nguyen H.D., Thi T.S.L. (2024). Clinical trial and real-world evidence of circulating tumor DNA monitoring to predict recurrence in patients with resected colorectal cancer. ESMO Real. World Data Digit. Oncol..

[64] Pan S., Wang G. (2026). Early postoperative hospital exposome stress, multisystem recovery trajectories, and long-term oncologic outcomes after curative colorectal cancer surgery. Surg. Oncol..

[65] Pan S., Wang G. (2026). Perioperative physiological complexity trajectories and their association with molecular residual disease and long-term outcomes after curative colorectal cancer surgery: A prospective cohort study. Surg. Endosc..

[66] Pellatt A.J., Bent A., Hornstein N., Parseghian C., Huey R., Raghav K., Morris V., Overman M., Morelli P., Willis J. (2025). Phase II Trial of TAS-102 in Colorectal Cancer Patients With Circulating Tumor DNA-Defined Minimal Residual Disease After Adjuvant Therapy: INTERCEPT-TT. JCO Precis. Oncol..

[67] Salimiaghdam N., Li E., Liang H. (2026). When Blood Speaks Before the Scan: The Role of ctDNA in Real-time CRC Care. Cancer Diagn. Progn..

[68] Sørensen E.E., Frydendahl A., Rasmussen M.H., Nordentoft I., Knudsen M., Henriksen T.V., Lindskrog S.V., Dyrskjøt L., Andersen C.L., Bramsen J.B. (2026). Molecular residual disease assessment in colorectal and bladder cancer by somatic structural variant analysis of cell-free DNA whole-genome sequencing data. J. Transl. Med..

[69] Tie J., Wang Y., Cohen J., Li L., Hong W., Christie M., Wong H.L., Kosmider S., Wong R., Thomson B. (2021). Circulating tumor DNA dynamics and recurrence risk in patients undergoing curative intent resection of colorectal cancer liver metastases: A prospective cohort study. PLoS Med..

[70] Weinberg B.A., Winslow E.R., Bayasi M., Krainock M.R., Olshan P.M., Billings P.R., Aleshin A. (2021). Early Detection of Circulating Tumor DNA Postoperatively Enables Discovery of Resectable Metastatic Disease in a Patient with Colon Cancer. Case Rep. Oncol..

[71] Diergaarde B., Young G., Hall D.W., Mazloom A., Costa G.L., Subramaniam S., Palomares M.R., Garces J., Baehner F.L., Schoen R.E. (2025). Circulating Tumor DNA as a Marker of Recurrence Risk in Stage III Colorectal Cancer: The α-CORRECT Study. J. Clin. Oncol..

[72] Henriksen T.V., Demuth C., Frydendahl A., Nors J., Nesic M., Rasmussen M.H., Reinert T., Larsen O.H., Jaensch C., Løve U.S. (2024). Unraveling the potential clinical utility of circulating tumor DNA detection in colorectal cancer—Evaluation in a nationwide Danish cohort. Ann. Oncol..

[73] Chan H.T., Nagayama S., Otaki M., Chin Y.M., Fukunaga Y., Ueno M., Nakamura Y., Low S.-K. (2023). Tumor-informed or tumor-agnostic circulating tumor DNA as a biomarker for risk of recurrence in resected colorectal cancer patients. Front. Oncol..

[74] Franken I.A., Ketelaars S.L.C., Rubio-Alarcón C., Diemen P.D., Spaapen T.O.M., Heilijgers F., Guimaraes M.d.S., van Nassau S.C.M.W., Schraa S.J., Mesker W.E. (2026). Cancer-associated fibroblast signature and SMAD4 mutation in resistance to adjuvant chemotherapy in stage III colon cancer patients. Eur. J. Cancer.

[75] Pan S., Wang G. (2026). Perioperative Systemic Stress Phenotypes Predict Immune Recovery Failure, Molecular Residual Disease, and Long-Term Outcomes After Colorectal Cancer Surgery: A Prospective Cohort Study. World J. Surg..

[76] Frydendahl A., Rasmussen M.H., Jensen S.Ø., Henriksen T.V., Demuth C., Diekema M., Ditzel H.J., Wen S.W.C., Pedersen J.S., Dyrskjøt L. (2024). Error-Corrected Deep Targeted Sequencing of Circulating Cell-Free DNA from Colorectal Cancer Patients for Sensitive Detection of Circulating Tumor DNA. Int. J. Mol. Sci..

[77] Fukumoto Y., Kataoka K., Muroi Y., Koba M., Ito K., Zhenxin R., Imada A., Jihyung S., Horio Y., Kuwahara R. (2026). Real-world experience of circulating tumor DNA testing in resectable colorectal cancer: A Japanese single-institution observational study. Int. J. Clin. Oncol..

[78] Lutfi A., Afghan M.K., Swed B., Kasi P.M. (2023). False-Positive Liquid Biopsy Assays Secondary to Overlapping Aberrant Methylation from Non-Cancer Disease States. Case Rep. Oncol..

[79] Martín-Arana J., Gimeno-Valiente F., Henriksen T.V., García-Micó B., Martínez-Castedo B., Gambardella V., Martínez-Ciarpaglini C., Palomar B., Huerta M., Camblor D.G. (2025). Whole-exome tumor-agnostic ctDNA analysis enhances minimal residual disease detection and reveals relapse mechanisms in localized colon cancer. Nat. Cancer.

[80] Nakamura Y., Tsukada Y., Matsuhashi N., Murano T., Shiozawa M., Takahashi Y., Oki E., Goto M., Kagawa Y., Kanazawa A. (2024). Colorectal Cancer Recurrence Prediction Using a Tissue-Free Epigenomic Minimal Residual Disease Assay. Clin. Cancer Res..

[81] Parikh A.R., Corcoran R.B. (2021). Plasma-only ctDNA-Guided MRD Detection in Patients with CRC—Response. Clin. Cancer Res..

[82] Sinicrope F.A., Segovia D., Sharma N., Alberts S.R., Hardin A., Rich T., Shi Q. (2026). Tissue-Free Circulating Tumor DNA Assay and Patient Outcome in a Phase III Trial of FOLFOX-Based Adjuvant Chemotherapy (Alliance N0147). J. Clin. Oncol..

[83] Slater S., Bryant A., Aresu M., Begum R., Chen H.-C., Peckitt C., Lazaro-Alcausi R., Carter P., Anandappa G., Khakoo S. (2024). Tissue-Free Liquid Biopsies Combining Genomic and Methylation Signals for Minimal Residual Disease Detection in Patients with Early Colorectal Cancer from the UK TRACC Part B Study. Clin. Cancer Res..

[84] Parikh A.R., Van Seventer E.E., Siravegna G., Hartwig A.V., Jaimovich A., He Y., Kanter K., Fish M.G., Fosbenner K.D., Miao B. (2021). Minimal Residual Disease Detection using a Plasma-only Circulating Tumor DNA Assay in Patients with Colorectal Cancer. Clin. Cancer Res..

[85] Lee J.Y., Mun C.W., Kim E.R., Park S.-G., Lee M.-Y. (2026). Translational Feasibility of a Plasmonic Microarray–Based Liquid Biopsy for KRAS Codon Mutation Detection across Tissue, Plasma, and Urine in Early Colorectal Cancer. npj Precis. Oncol..

[86] Bregni G., Pretta A., Senti C., Reina E.A., Vandeputte C., Trevisi E., Gkolfakis P., Kehagias P., Laethem J.-L.V., Vergauwe P. (2022). Circulating DNA in the neoadjuvant setting of early stage colon cancer. Acta Oncol..

[87] do Canto L.M., Raunkilde L., Lindebjerg J., Aagaard M.M., Therkildsen C., Kleif J., Jensen L.H., Hansen T.F., Andersen R.F. (2026). Detection of circulating tumor DNA in colorectal cancer patients using a methylation-specific droplet digital PCR multiplex. Mol. Oncol..

[88] Gong J., Aguirre F., Hazelett D., Alvarez R., Zhou L., Hendifar A., Osipov A., Zaghiyan K., Cho M., Gangi A. (2022). Circulating tumor DNA dynamics and response to immunotherapy in colorectal cancer. Mol. Clin. Oncol..

[89] Nguyen T., Nguyen Hoang V.-A., Nguyen T.H., Tran T.H., Nguyen N., Le Vo T.T., Nguyen D.S., Giang H., Nguyen H.-N., Tu L.N. (2025). Tumor-naïve multimodal profiling of circulating tumor DNA to detect minimal residual disease in solid tumors. Ther. Adv. Med. Oncol..

[90] Baraniskin A., Baba H.A., Theegarten D., Mika T., Schroers R., Klein-Scory S. (2023). Liquid biopsy can cure early colorectal cancer recurrence—Case Report. Front. Oncol..

[91] Boysen A.K., Pallisgaard N., Andersen C.S.A., Spindler K.-L.G. (2020). Circulating tumor DNA as a marker of minimal residual disease following local treatment of metastases from colorectal cancer. Acta Oncol..

[92] Ganesan A., Korkiakoski A., Adamusová S., Musku A., Rantasalo T., Laine N., Andersson E., Osterlund E., Ovissi A., Halonen P. (2025). Evaluation of Bridge Capture technology for mutation profiling in liquid biopsies of metastatic colorectal cancer patients. Sci. Rep..

[93] Gimeno-Valiente F., Martín-Arana J., Tébar-Martínez R., Gambardella V., Martínez-Ciarpaglini C., García-Micó B., Martínez-Castedo B., Palomar B., García-Bartolomé M., Seguí V. (2023). Sequencing paired tumor DNA and white blood cells improves circulating tumor DNA tracking and detects pathogenic germline variants in localized colon cancer. ESMO Open.

[94] Iwai T., Yamada T., Uehara K., Shinji S., Matsuda A., Yokoyama Y., Takahashi G., Miyasaka T., Yoshida H. (2025). Clinical Implications of Cell-Free DNA in Managing BRAF V600E Mutation-Positive Colorectal Cancer. Genes.

[95] Kanaka S., Yamada T., Asai F., Shimizu Y., Uehara K., Matsuda A., Shinji S., Yokoyama Y., Takahashi G., Iwai T. (2026). Utility of Urinary Extracellular Vesicles for Colorectal Cancer: Comparison of DNA Quality and MRD Detection. Cancer Sci..

[96] Mögele T., Höck M., Sommer F., Friedrich L., Sommer S., Schmutz M., Altenburger A., Messmann H., Anthuber M., Kröncke T. (2024). Circulating Tumor DNA for Prediction of Complete Pathological Response to Neoadjuvant Radiochemotherapy in Locally Advanced Rectal Cancer (NEORECT Trial). Cancers.

[97] Nakano T., Takao S., Dairaku K., Uno N., Low S.-K., Hashimoto M., Tsuda Y., Hisamatsu Y., Toshima T., Yonemura Y. (2024). Implementable assay for monitoring minimum residual disease after radical treatment for colorectal cancer. Cancer Sci..

[98] Ohta R., Yamada T., Sonoda H., Matsuda A., Shinji S., Takahashi G., Iwai T., Takeda K., Ueda K., Kuriyama S. (2021). Detection of KRAS mutations in circulating tumour DNA from plasma and urine of patients with colorectal cancer. Eur. J. Surg. Oncol..

[99] Reinert T., Henriksen T.V., Aleshin A., Zimmermann B., Andersen C.L. (2021). Plasma-only ctDNA-Guided MRD Detection in Patients with CRC—Letter. Clin. Cancer Res..

[100] Siravegna G., Sartore-Bianchi A., Mussolin B., Cassingena A., Amatu A., Novara L., Buscarino M., Corti G., Crisafulli G., Bartolini A. (2017). Tracking aCAD-ALK gene rearrangement in urine and blood of a colorectal cancer patient treated with an ALK inhibitor. Ann. Oncol..

[101] Tarazona N., Gimeno-Valiente F., Gambardella V., Huerta M., Roselló S., Zuniga S., Calon A., Carbonell-Asins J.A., Fontana E., Martinez-Ciarpaglini C. (2020). Detection of postoperative plasma circulating tumour DNA and lack of CDX2 expression as markers of recurrence in patients with localised colon cancer. ESMO Open.

[102] Tarazona N., Gimeno-Valiente F., Gambardella V., Zuñiga S., Rentero-Garrido P., Huerta M., Roselló S., Martinez-Ciarpaglini C., Carbonell-Asins J.A., Carrasco F. (2019). Targeted next-generation sequencing of circulating-tumor DNA for tracking minimal residual disease in localized colon cancer. Ann. Oncol..

[103] Miyazaki K., Montcusí B., Morine Y., Okuno K., Pascual M., Tokunaga T., Noma T., Kinugasa Y., Shimada M., Cai G. (2026). An exosome-based liquid biopsy to predict molecular residual disease for the identification of high-risk patients with stage II-III colorectal cancer: The CLEAR study. Mol. Cancer.

[104] Kasi P.M., Afghan M.K., Bellizzi A.M., Chan C.H. (2022). Larotrectinib in Mismatch-Repair-Deficient TRK Fusion-Positive Metastatic Colon Cancer After Progression on Immunotherapy. Cureus.

[105] Luo J., Zhou B., Zhao L., Yuan J., Zhou J., Shen L., Li F., Qian C. (2023). Circulating tumor DNA analysis detects micrometastatic disease and predicts recurrence in a patient with colon cancer: A case report. Medicine.

[106] Mallampati S., Zalles S., Duose D.Y., Hu P.C., Medeiros L.J., Wistuba I.I., Kopetz S., Luthra R. (2019). Development and Application of Duplex Sequencing Strategy for Cell-Free DNA–Based Longitudinal Monitoring of Stage IV Colorectal Cancer. J. Mol. Diagn..

[107] Lin Z., Zhai M., Wang H., Li M., Liu L., Zhang P., Yan L., Liu H., Tao K., Zhang T. (2025). Longitudinal Circulating Tumor DNA Monitoring in Predicting Response to Short-Course Radiotherapy Followed by Neoadjuvant Chemotherapy and Camrelizumab in Locally Advanced Rectal Cancer: Data from a Phase III Clinical Trial (UNION). Cancer Lett..

[108] Zhang X., Feng R., Xu Y., Yang L., Xie F., Yang H., Wang S., Peng Y., Liu M., Wang C. (2024). Baseline circulating tumor DNA predicts long-term survival outcomes for patients with early breast cancer. Gland Surg..

[109] Liu B., Zhou H., Tan L., Siu K.T.H., Guan X.-Y. (2024). Exploring treatment options in cancer: Tumor treatment strategies. Signal Transduct. Target. Ther..

[110] Slater S., Bryant A., Chen H.-C., Begum R., Rana I., Aresu M., Peckitt C., Zhitkov O., Lazaro-Alcausi R., Borja V. (2023). ctDNA guided adjuvant chemotherapy versus standard of care adjuvant chemotherapy after curative surgery in patients with high risk stage II or stage III colorectal cancer: A multi-centre, prospective, randomised control trial (TRACC Part C). BMC Cancer.

[111] Wang Y., Yang L., Bao H., Fan X., Xia F., Wan J., Shen L., Guan Y., Bao H., Wu X. (2021). Utility of ctDNA in predicting response to neoadjuvant chemoradiotherapy and prognosis assessment in locally advanced rectal cancer: A prospective cohort study. PLoS Med..

[112] Srivastava R., Lodhi N. (2022). DNA Methylation Malleability and Dysregulation in Cancer Progression: Understanding the Role of PARP1. Biomolecules.

[113] Li L., Sun Y. (2024). Circulating tumor DNA methylation detection as biomarker and its application in tumor liquid biopsy: Advances and challenges. MedComm.

