# Supplementary material for: Circulating Tumor DNA (ctDNA) for Minimal Residual Disease (MRD) Detection in Colorectal Cancer (CRC): Current Evidence, Clinical Applications, and Future Directions
Source: Curr Oncol. 2026 Oct 9;33(10):606. doi: 10.3390/curroncol33100606 (PMC13703757; doi:10.3390/curroncol33100606)
Supplement: Supplementary file 1 [file curroncol-33-00606-s001.zip › curroncol-4547809- supplementary.pdf]

Supplementary Data

Supplementary Table S1. Study characteristics, ctDNA methodologies, phase-specific clinical performance, prognostic outcomes, and clinical utility of publications evaluating minimal residual disease in colorectal cancer. The table summarizes the 94 publications included in the structured literature review and separately identifies additional pivotal randomized evidence incorporated during peer review. Analytical performance and detection thresholds are presented separately from preoperative or baseline clinical findings, postoperative landmark testing, post-treatment or end-of-treatment testing, and longitudinal surveillance. Preoperative ctDNA detection rates are labelled as positivity or detection rates and are not interpreted as minimal residual disease sensitivity. Sensitivity and specificity are presented separately from hazard ratios, recurrence outcomes, ctDNA clearance or persistence, and treatment-guidance endpoints. "Not assessed" indicates that the publication did not evaluate the specified clinical phase; "NR" indicates that the measure was relevant but not reported or not extractable from the available study information. Publications derived from overlapping cohorts may include shared participants and should not be interpreted as independent patient datasets. Abbreviations: ACT, adjuvant chemotherapy; CEA, carcinoembryonic antigen; cfDNA, cell-free DNA; CI, confidence interval; CRC, colorectal cancer; ctDNA, circulating tumor DNA; DFS, disease-free survival; dPCR, digital polymerase chain reaction; ddPCR, droplet digital polymerase chain reaction; EOT, end of treatment; HR, hazard ratio; MRD, minimal residual disease; NGS, next-generation sequencing; NPV, negative predictive value; OS, overall survival; PCR, polymerase chain reaction; PPV, positive predictive value; RFS, recurrence-free survival; SNV, single-nucleotide variant; VAF, variant allele frequency; WES, whole-exome sequencing; WGS, whole-genome sequencing.

Study (First Author, Year)	DOI	Study design / trial	Population	Treatment context	Biomarker(s) / target(s)	Analytical performance / detection threshold	Preoperative / baseline findings	Postoperative landmark findings	Post-treatment / EOT findings	Longitudinal surveillance findings	Phase-Specific Prognostic Outcomes / ctDNA Dynamics	Clinical utility endpoints	Assay methodology

Alden, S. et al, 2022 [46]	10.3389/fonc.2022.1074786	Case report - descriptive evidence only	Age/sex: 57<br>Region: United States<br>Cohort: 1 stage I colon cancer	Systemic treatment: five cycles of leucovorin, 5-fluorouracil, oxaliplatin, and irinotecan with bevacizumab (FOLFOXIRI-Bev)	ctDNA	Not assessed	Not assessed	Not assessed	Not assessed	Descriptive longitudinal monitoring; no comparative performance estimate.	NR	NR	tumor-informed, multiplex PCR (mPCR) next-generation sequencing (NGS)-based assay (Signatera™, Natera, Inc.). whole-exome sequencing (WES).
Andersen L. et al, 2024 [34]	10.1016/j.ejca.2024.114186	Clinical cohort; prospective/retrospective design NR	Age/sex: 63 - 78 years (-ve - 70 years, +ve - 72 years)<br>Region: Treated at Aarhus University Hospital, Denmark<br>Cohort: 747 652 WES 95 primary tumor RNA sequencing	NR	MSI ctDNA-patient-specific mutations	Clinical performance reported; assessment phase NR. Specificity: True negative patients: 261, False positive patients: 21	NR	NR	NR	NR	NR	NR	1.Droplet Digital PCR (ddPCR), Target patient-specific clonal mutation, single somatic structural variant, or TriMeth assay 2. Deep Targeted Sequencing, 3.Signatera ultradeep multiplex PCR sequencing
Ando, K. et al, 2026 [47]	10.1097/SLA.0000000000006948	Clinical cohort; prospective/retrospective design NR	Age/sex: 38-92 (median = 67)<br>Region: Japan<br>Cohort: 250 patients with stage II-III rectal cancer	Adjuvant treatment: Adjuvant chemotherapy (ACT)	Postoperative ctDNA	NR	NR	NR	NR	NR	NR	NR	tumor-informed 16-plex PCR next-generation sequencing assay (Signatera)
Azzi, G. et al, 2025 [48]	10.1159/000547249	Case report - descriptive evidence only	Age/sex: 53<br>Region: United States<br>Cohort: 1 Stage III Rectal Cancer patient	folinic acid, fluorouracil, oxaliplatin, and irinotecan (FOLFOXIRI)	ctDNA	Not assessed	Not assessed	Not assessed	Not assessed	Descriptive longitudinal monitoring; no comparative performance estimate.	NR	NR	commercial tumor-informed ctDNA MRD assay, advanced (Haystack) MRD assay

Bando, H. et al, 2026 [18]	10.1038/s41591-026-04428-0	Randomized phase III trial - ALTAIR	Age/sex: < 70 years of age (n = 155, 63.8%) Region: Japan and Taiwan Cohort: 243 patients with stage 0-IV CRC	Adjuvant treatment: Adjuvant chemotherapy (ACT), trifluridine/tipiracil hydrochloride therapy	ctDNA (Up to 16 somatic single-nucleotide variants (SNVs) were selected)	Not assessed	Not assessed	Not assessed	Molecular recurrence detected after completion of standard therapy; all randomized patients were ctDNA-positive without radiologic disease.	ctDNA surveillance identified molecular recurrence and triggered trial enrollment.	Median DFS: 9.30 months with trifluridine/tipiracil vs 5.55 months with placebo; HR 0.79 (95% CI 0.60-1.05), P=0.107.	ALTAIR phase III: trifluridine/tipiracil did not significantly improve DFS compared with placebo; the primary endpoint was not met and hematologic toxicity was greater.	whole-exome sequencing (WES). tumor-informed 16-plex polymerase chain reaction (PCR) next-generation sequencing (NGS) assay (Signatera; Natera) of plasma cell free DNA
Baraniskin A. et al, 2023 [90]	10.3389/fonc.2023.1141833	Case report - descriptive evidence only	Age/sex: 47 year old Region: Germany Cohort: 1	Systemic treatment: FOLFOX4 (a combination of folic acid, fluorouracil, and oxaliplatin) and bevacizumab	NRAS (NRAS G12D c.35G>A), TP53 C141Y c.422G>A)	Not assessed	Not assessed	Not assessed	Not assessed	Descriptive longitudinal monitoring; no comparative performance estimate.	NR	NR	ddPCR
Benhaim L. et al, 2021 [35]	10.1016/j.ejca.2021.09.004	Prospective multicentre cohort - ALGECOLS	Age/sex: 30.1 - 86.1 (mean = 66.7) Region: France Cohort: 187 (3 without plasma)	NR	WIF1, NPY, ctDNA patient-specific mutations	NR	Preoperative ctDNA positivity: 27.5% (positivity, not MRD sensitivity). Reported assay performance at D0: sensitivity 50%; specificity 77%.	Immediate postoperative (D5): sensitivity 27%; specificity 93%.	Not assessed	Early postoperative follow-up (1-6 months): serial ctDNA assessment; formal sensitivity/specificity NR.	Recurrence: 32.7% with detectable preoperative ctDNA vs 11.6% without. Time to recurrence: preoperative adjusted HR 3.58 (95% CI 1.71-7.47); immediate postoperative HR 3.22 (95% CI 1.32-7.89); early	NR	ddPCR, targeting mutation & methylation markers

											follow-up HR 5.00 (95% CI 1.90-12.90).		
Bikhchandani, M. et al, 2023 [49]	10.3390/genes14051054	Case report - descriptive evidence only	Age/sex: 55<br>Region: United States<br>Cohort: 1 stage IIIB colon cancer	Adjuvant treatment: treated with twelve cycles of adjuvant 5-fluorouracil, leucovorin, and oxaliplatin (FOLFOX). pembrolizumab	ctDNA, POLE P436R mutation	Not assessed	Not assessed	Not assessed	Not assessed	Descriptive longitudinal monitoring; no comparative performance estimate.	NR	NR	Next-generation sequencing (NGS), ctDNA assay (Signatera), Germline testing (Invitae 91-gene panel)
Boysen A. et al, 2020 [91]	10.1080/0284186X.2020.1806357	Clinical cohort; prospective/retrospective design NR	Age/sex: Older than 18<br>Region: Aarhus University Hospital, Denmark<br>Cohort: 35	Local/curative-intent treatment: Surgery, Radiofrequency Ablation (RFA) & Stereotactic Body Radiotherapy (SBRT)	KRAS, NRAS, BRAF	Analytical performance (not a clinical MRD phase). Sensitivity/detection: 49% ddPCR with limit of detection of 0.1%	NR	Not assessed	Not assessed	Not assessed	NR	NR	ddPCR
Bregni, G. et al, 2022 [86]	10.1080/0284186x.2022.2101023	Clinical cohort; prospective/retrospective design NR	Age/sex: median = 63<br>Region: Belgium<br>Cohort: 80 stage III colon cancer patients	Perioperative treatment: neoadjuvant and adjuvant FOLFOX chemotherapy	ctDNA (NPY and WIF1 methylation)	NR	NR	NR	NR	NR	NR	NR	ddPCR
Burley, N. et al, 2024 [50]	10.1080/1750743X.2024.2430941	Case series - descriptive evidence only	Age/sex: 43, 54<br>Region: United States<br>Cohort: 2	Adjuvant treatment: surgery followed by traditional adjuvant combination chemotherapy, then switched to pembrolizumab	ctDNA	Not assessed	Not assessed	Not assessed	Not assessed	Descriptive longitudinal monitoring; no comparative performance estimate.	NR	NR	tumor-informed ctDNA assay (Signatera)
Chakrabarti, S. et al, 2026 [51]	10.3390/cancers18040589	Clinical cohort; prospective/retrospective design NR	Age/sex: 32-89 (median = 59)<br>Region: United States<br>Cohort: 220 stage II-III rectal cancer patients	Perioperative treatment: total neoadjuvant therapy (TNT), chemotherapy alone, and chemoradiation/radiation alone.	ctDNA	NR	NR	NR	NR	NR	NR	NR	personalized tumor-informed assay (Signatera), 16-plex PCR next-generation sequencing (NGS) assay, whole-exome sequencing (WES), multiplex PCR (mPCR)

Chan H. et al, 2023 [73]	10.3389/fonc.2022.1055968	Clinical cohort; prospective/retrospective design NR	Age/sex: 42 - 88 (median = 66) Region: Tokyo Cohort: 38	NR	ctDNA, TP53, KRAS, PIK3CA, APC and VAF	Analytical VAF range: 0.018%-0.783%.	NR	Landmark recurrence detection: sensitivity 67%; specificity 87%.	Not assessed	Longitudinal recurrence detection: sensitivity 100%; specificity 84%.	NR	NR	Qiagen for extraction and purification NGS using Oncomine Pan Cancer Cell-Free Assay, DNA Extraction, Sequencing Data Analysis by using Torrent Suite Software and Ion Reporter, Statistical Analysis performed using Kaplan-Meier method and Cox proportional hazards regression analysis.
Chen G. et al, 2021 [44]	10.1186/s13045-021-01089-z	Clinical cohort; prospective/retrospective design NR	Age/sex: 19 - 84 (median = 60) Region: China Cohort: 2240 stage I, II, III CRC	Adjuvant treatment: Adjuvant chemotherapy (ACT)	SMAD4, PTEN, and PKHD1	Analytical VAF threshold: 0.01%.	Preoperative ctDNA detection/positivity (not MRD sensitivity): 64.2% overall; 65.2% stage II; 63.3% stage III.	Postoperative (day 3-7): sensitivity 82.6%; specificity 91.7%.	Post-adjuvant therapy: specificity 94.1%; sensitivity NR.	Not assessed	NR	NR	Targeted sequencing, Variant Identification, ctDNA detection
Diergaarde, B. et al, 2025 [71]	10.1002/jso.27989	Stage III cohort - alpha-CORRECT	Age/sex: mean (SD) = 64.5 (10.8) Region: United States Cohort: 124 patients with stage III CRC (118 colon cancer, 6 rectal cancer)	Adjuvant treatment: surgery followed by adjuvant chemotherapy (ACT) (96%), with (92%) receiving platinum-based chemotherapy	ctDNA	Not assessed	Not assessed	Postsurgical timepoint: sensitivity 77.8% (95% CI 54.8%-91.0%); specificity 74.2% (95% CI 62.6%-83.3%).	Post-definitive therapy: performance estimates NR in extraction table.	Surveillance: formal sensitivity/specificity NR.	Recurrence risk: postsurgical HR 9.6 (95% CI 3.2-29.5); post-definitive therapy HR 16.7 (95% CI 6.9-40.3); surveillance HR 49.6 (95% CI 16.6-148.3).	NR	whole-exome sequencing (WES) followed by tumor informed assays, ctDNA status was determined using plasma cell-free DNA.

											Three-year RFS, ctDNA-positive vs negative: 54.5% vs 96.1% postsurgery; 18.2% vs 90.0% post-definitive therapy.		
do Canto, L.M. et al, 2026 [87]	10.1002/1878-0261.70161	Assay development/validation cohort	Age/sex: Localized CRC: 41-96 (median = 73)<br>Region: Denmark<br>Cohort: 59 patients with colon or rectal cancer stage I-III (plasma samples for assay cutoff and sensitivity, localized CRC)	Adjuvant treatment: Previous adjuvant chemotherapy for radical treatment of stage II or III colorectal cancer was allowed in patients proven disease-free for more than 6 months.	methylated ctDNA	Clinical performance reported; assessment phase NR.<br>Sensitivity/detection: 64.4% (localized), 89.2% (mCRC)<br>Specificity: 96.70%	NR	NR	NR	NR	NR	NR	methylation specific droplet digital PCR (MS-ddPCR) multiplex assay
Emiloju, O. E. et al, 2024 [52]	10.1200/PO.23.00127	Clinical cohort; prospective/retrospective design NR	Age/sex: median = 67<br>Region: United States<br>Cohort: 120 patients CRC stage I-IV	NR	ctDNA	NR	NR	NR	NR	NR	NR	NR	tumor-informed ctDNA assay (Signatera)
Eslinger C. et al, 2024 [9]	10.21037/jgo-24-81	Case report - descriptive evidence only	Age/sex: 75 year old (male)<br>Region: Phoenix, Arizona<br>Cohort: 1	Systemic treatment: Pembrolizumab	MSH6, MLH1, MSH2, MSH6, PMS2, ACVR2A, APC, B2M, CDK12, CDKN2A, FANCE, FBXW7, KEAP1, KMT2B, KMT2C*, MAP3K1, MSH3, PIK3CA, PIK3R1, RASA1, SMARCA4, SOX9, TCF7L2, TSC2, ARID1A, ARID2, ASXL1, ATM, EP300, ERBB2, ERBB3, GNAS, HRAS, RNF43,	Not assessed	Not assessed	Not assessed	Not assessed	Descriptive longitudinal monitoring; no comparative performance estimate.	NR	NR	Tumor-informed assays Natera/Signatera

					GLYR1, KMT2C* & ZBTB7A.								
Fan W. et al, 2024 [42]	10.1177/17588359231220607	Clinical cohort; prospective/retrospective design NR	Age/sex: 18 years & older<br>Region: China<br>Cohort: 309 Stage I, II, III, IV CRC	NR	ZFP36L2, GAB2, CTNNB1, MLL3 & ATR Personalized ctDNA markers	Not assessed	Not assessed	Postsurgery MRD: sensitivity 64.6%; specificity 94.8%; PPV 75.6%; NPV 91.5%.	Not assessed	Longitudinal MRD: sensitivity 75.0%; specificity 94.9%; PPV 79.6%; NPV 93.4%.	Recurrence risk: postoperative HR 13.17 (95% CI NR); longitudinal HR 14.44 (95% CI NR). Detection was less effective for lung-only and peritoneal recurrence.	Adjuvant therapy did not confer a survival advantage within detectable or undetectable MRD subgroups in this observational analysis.	NGS & ctDNA based MRD assay that integrated NGS genotype information with tumor-naive ctDNA
Folprecht, G. et al, 2026 [19]	10.1016/j.annonc.2026.05.001	Randomized trial - CIRCULATE AIO/ABCSG	Age/sex: median age = 64<br>Region: Germany and Austria<br>Cohort: 1722 patients (ctDNA analyzed) stage II colon cancer or rectal cancer ctDNA positive: 55 ctDNA negative: 1667 1396 randomized patients (OFF-STUDY, OBS and CHEMO)	capecitabine ± oxaliplatin	ctDNA	Not assessed	Not assessed	Postoperative sample (median 21 days): sensitivity 31% (95% CI 19%-44%); specificity 98% (95% CI 95%-99%); PPV 72%; NPV 88%. Median ctDNA VAF 0.089%.	NR	NR	ctDNA-positive vs negative: 3-year DFS 52% vs 87%, HR 4.28 (95% CI 2.32-7.93); 3-year OS 88% vs 98%, HR 5.48 (95% CI 1.64-18.28).	ITT: chemotherapy vs observation did not significantly improve recurrence or DFS (DFS HR 0.55, 95% CI 0.21-1.48). Per-protocol analysis suggested fewer recurrences (HR 0.23, 95% CI 0.06-0.87) and improved DFS (HR 0.31, 95% CI 0.09-1.03). Trial closed early and was underpowered.	tumour-informed ctDNA assay, next-generation sequencing (NGS) of plasma cell free DNA using up to three patient-specific somatic mutations

Franken, I. A. et al, 2026 [53]	10.1016/j.esmoop.2025.105935	Clinical cohort; prospective/retrospective design NR	Age/sex: mean = 63.1 Region: Netherlands Cohort: 206 stage III colon cancer patients	Adjuvant treatment: Adjuvant chemotherapy (ACT) (CAPOX or capecitabine)	ctDNA	NR	NR	NR	NR	NR	NR	NR	tumor tissue-informed ctDNA assay (Labcorp® Plasma Detect™) involving integrated whole genome sequencing analyses of plasma cell-free DNA, germline DNA and tumor tissue DNA.
Franken, I. et al, 2026 [74]	10.1016/j.ejca.2026.116262	Clinical cohort; prospective/retrospective design NR	Age/sex: mean = 62.2 Region: Netherlands Cohort: 259 stage III colon cancer patients	Perioperative treatment: adjuvant CAPOX or capecitabine, and no neoadjuvant treatment	ctDNA, SMAD4, CAF signature	NR	NR	NR	NR	NR	NR	NR	PGDx® elio™ tissue complete assay, ctDNA analyses on plasma (Labcorp® Plasma Detect™), RNA sequencing
Frydendahl A. et al, 2024 [76]	10.3390/ijms25084252	Assay development/validation cohort	Age/sex: NR Region: NR Cohort: 364 249 CRC patients 37 healthy patients	NR	Compared healthy patient ctDNA to patients with CRC and found differences such as single-nucleotide variants (SNV), Insertions and Deletions (INDELs), multi-nucleotide variants (MNVs)	Clinical performance reported; assessment phase NR. Sensitivity/detection: Sensitivity of UMIseq was found to be 80% in training cohort & 70% in the validation cohort Specificity: Specificity of UMIseq was found to be 95%	NR	NR	NR	NR	NR	NR	UMIseq fixed-panel deep targeted sequencing approach ddPCR
Frydendahl, A. et al, 2025 [54]	10.1002/1878-0261.13803	Assay validation cohort	Age/sex: 60-72 (median = 66) Region: Denmark Cohort: cohort 1: 93 patients with stage III colorectal cancer (CRC)	NR	ctDNA	Clinical performance reported; assessment phase NR. Sensitivity/detection: 96% Specificity: 95%	NR	NR	NR	NR	NR	NR	WGS of tumor DNA, normal DNA from WBCs, and plasma cfDNA using the NovaSeq

			and 40 healthy individuals										platform, tumor-informed ctDNA detection of patient specific somatic SNVs (MRD-EDGESNV)
Fukumoto, Y. et al, 2026 [77]	10.1007/s10147-025-02943-7	Real-world observational cohort	Age/sex: 33-84 (median = 69)<br>Region: Japan<br>Cohort: 56 CRC patients (3 patients censored due to external follow-up)	CAPOX (capecitabine + oxaliplatin), capecitabine monotherapy	ctDNA methylation markers	NR	NR	NR	NR	NR	NR	NR	tissue-agnostic guardant reveal assay, next-generation sequencing (NGS) to detect differentially methylated regions (DMR)
Ganesan, A. et al, 2025 [92]	10.1038/s41598-025-04827-2	Clinical cohort; prospective/retrospective design NR	Age/sex: NR<br>Region: Finland<br>Cohort: 10 metastatic CRC patients	NR	ctDNA, KRAS codon 12 or 13 mutation (G12D, G12V, G12C, G13D, or G13R)	Clinical performance reported; assessment phase NR. Sensitivity/detection: NR; VAF: median 0.03%; range 0-7.74%	NR	NR	NR	NR	NR	NR	NGS-based Bridge Capture, ddPCR with validated mutation detection assays for KRAS, real-time PCR-based Idylla ctKRAS Mutation Assay
Gao Z. et al, 2023 [55]	10.1186/s12967-023-03884-3	Development and validation cohort	Age/sex: 29 - 75 (median = 61)<br>Region: Beijing, China<br>Cohort: 249 124- the discovery cohort Stage I, II, III CRC	NR	Patient-specific ctDNA mutations	Analytical performance (not a clinical MRD phase). Sensitivity/detection: Predicting recurrence after 24 months was found to be 49.6% VAF thresholds from 0.02-0.05% Specificity: Predicting recurrence after 24 months was found to be 86.5%	NR	Not assessed	Not assessed	Not assessed	NR	NR	733-gene panel for tumor tissue -NGS and a 127-gene panel for circulating tumor DNA (ctDNA) detection

Gimeno - Valiente , F. et al, 2023 [93]	10.1016/j.esmoop.2023.102051	Clinical cohort; prospective/retrospective design NR	Age/sex: NR Region: Spain Cohort: 148 localized colon cancer stage I-III patients	NR	ctDNA	Analytical performance (not a clinical MRD phase). Sensitivity/detection: 53% primary tumor variants, VAF threshold of 5% was applied, while for plasma samples it was set at 0.01%	NR	Not assessed	Not assessed	Not assessed	NR	NR	Droplet digital PCR and amplicon-based NGS. custom 29-gene panel, sequencing was conducted on tumor tissue, plasma and corresponding WBCs
Gong J. et al, 2022 [88]	10.3892/mco.2022.2533	Case series - descriptive evidence only	Age/sex: 55 - 74 Region: United States Cohort: 4	Systemic treatment: Regorafenib, Pembrolizumab, & Nivolumab	ctDNA & mSEPT9 (SEPTIN9)	Not assessed	Not assessed	Not assessed	Not assessed	Descriptive longitudinal monitoring; no comparative performance estimate.	NR	NR	modified Epi proColon® 2.0 CE assay for ctDNA testing on the methylated SEPTIN9 gene (mSEPT9)
Henriksen T. et al, 2022 [27]	10.1158/1078-0432.CCR-21-2404	Prospective clinical cohort	Age/sex: 18 years and older Region: Danish Cohort: 168 Stage III CRC	Adjuvant treatment: adjuvant chemotherapy (ACT) treatment used was fluoropyrimidine combined with oxaliplatin	patient-specific somatic single-nucleotide variants (SNVs)	Not assessed	Not assessed	Postoperative ctDNA: reported sensitivity 80%; specificity reported as 80%-95% in the extraction table.	Immediately after adjuvant chemotherapy: only patients with permanent ctDNA clearance during therapy remained recurrence-free.	Serial surveillance after treatment: median lead time 9.8 months versus computed tomography.	Postoperative recurrence HR 7.0 (95% CI 3.7-13.5). Immediately post-ACT recurrence HR 50.76 (95% CI 15.4-167). Serial-surveillance recurrence HR 50.80 (95% CI 14.9-172). Postoperative ctDNA-positive patients treated with ACT	NR	multiplex-PCR and next-generation sequencing to profile patient-specific somatic single-nucleotide variants via WES and Signatera

											had an 80% recurrence rate. ctDNA growth rate was associated with survival (HR 2.7, 95% CI 1.1-6.7).		
Henriksen T. et al, 2024 [72]	10.1016/j.annonc.2023.11.009	Nationwide clinical cohort	Age/sex: 64 - 77 (median - 71)<br>Region: Danish<br>Cohort: 851 stage II and stage III CRC	NR	patient-specific clonal mutations	Not assessed	Not assessed	Sensitivity/detection: sensitivity of post-operative ctDNA detection was 35%<br>Specificity: specificity of post-operative ctDNA detection was 98%	Not assessed	Not assessed	NR	NR	digital PCR (dPCR) WES
Hisamatsu, Y. et al, 2025 [56]	10.1111/cas.70114	Clinical cohort; prospective/retrospective design NR	Age/sex: 31-91 (median = 69.5)<br>Region: Japan<br>Cohort: 43 patients stage II CRC	capecitabine plus oxaliplatin, fluorouracil plus leucovorin plus oxaliplatin, or capecitabine	somatic ctDNA variants in 14 genes: AKT1, APC, BRAF, CTNNB1, ERBB3, FBXW7, KRAS, NRAS, PIK3CA, POLE, PPP2R1A, RNF43, SMAD4, and TP53	Clinical performance reported; assessment phase NR.<br>Sensitivity/detection: tumour informed assay: 25% plasma informed assay: 67% tumor-naive: 75%<br>Specificity: tumour informed assay: 91% plasma informed assay: 75% tumor-naive: 63%	NR	NR	NR	NR	NR	NR	Sysmex PSS CRC Assay
Iwai, T. et al, 2025 [94]	10.3390/genes16030275	Clinical/assay cohort; design details as reported	Age/sex: cohort 1: 59.0-71.5 (median = 69) cohort 2: 64.5-82.5 (median = 72)<br>Region: Japan<br>Cohort: 37 patients, 14 were Stage IV (cohort	Adjuvant treatment: FOLFOXIRI + Bevacizumab, mFOLFOX6 + Bevacizumab, and FOLFOX6. adjuvant chemotherapy (regimens: CAPOX:5,	cfDNA BRAFV600E mutation	Analytical performance (not a clinical MRD phase).<br>Sensitivity/detection: 57.1% VAF detection threshold of ≥0.1%<br>Specificity: 100%	NR	Not assessed	Not assessed	Not assessed	NR	NR	Droplet Digital PCR (ddPCR)

			1), 23 were Stage I-III (cohort 2)	FOLFOX:1, UFT/UZEL:3)									
Jain S. et al, 2024 [17]	10.3389/fonc.2024.1426941	Clinical cohort; prospective/retrospective design NR	Age/sex: 17 - 90 (median - 59)<br>Region: Asian countries<br>Cohort: 215 patients with 279 samples	NR	ctDNA	Not assessed	Not assessed	Not assessed	Not assessed	Sensitivity/detection: current version of the assay reported 81% sensitivity with serial samples for colon cancer<br>Specificity: Post-treatment sample level specificity of 98.2%	NR	NR	Next-generation sequencing (NGS) Guardant Reveal assay= MRD assay
Jiang H. et al, 2023 [14]	10.3389/fonc.2023.1153685	Clinical cohort; prospective/retrospective design NR	Age/sex: NR<br>Region: China<br>Cohort: 67	NR	AKT1, APC, BRAF, CTNB1, EGFR, ERBB2, FBXW7, GNAS, KRAS, MAP2K1, NRAS, PIK3CA, SMAD4, TP53	Not assessed	Not assessed	Sensitivity/detection: sensitivity of the tumor-naive strategy for detecting postoperative molecular residual disease (MRD) was 37%<br>Specificity: Thought to decrease as sensitivity increases	Not assessed	Not assessed	NR	NR	Next-generation sequencing (NGS) panels containing 14 colorectal cancer hotspot mutated genes (tumor-naive) Illumina
Kalil J. et al, 2024 [21]	10.3389/fonc.2024.1418696	Clinical cohort; prospective/retrospective design NR	Age/sex: 44 - 80 (median - 61)<br>Region: Canada<br>Cohort: 29	Perioperative treatment: Neoadjuvant Chemotherapy	KRAS (codons G12, G13, Q61)and PIK3CA (E542K, E545K, Q546K, H1047R, H1047L) mutations	NR	At liver resection: ctDNA detectable in 16/29; this is positivity at resection, not MRD sensitivity.	Initial postoperative sample (1-3 months): ctDNA detected in 8/29; all 8 recurred. Among ctDNA-negative patients, 11/21	Not assessed	Serial blood collection every 3 months; formal sensitivity/specificity NR.	At resection, recurrence occurred in 81% with detectable ctDNA vs 46% without. Postoperative DFS: 9	NR	Naica System Crystal Digital PCR (dPCR)

								(52%) recurred.			vs 13 months; HR 2.95 (95% CI 1.16-7.49).		
Kanaka, S. et al, 2026 [95]	10.1111/cas.70368	Clinical/assay cohort; design details as reported	Age/sex: Quality-validation cohort: mean = 67.4 survival-analysis cohort (positive group N=20): mean = 69 survival-analysis cohort (negative group N=243): mean = 67.9<br>Region: Japan<br>Cohort: 97 (Quality-validation cohort) CRC patients 63 (survival-analysis cohort) CRC patients	Adjuvant treatment: adjuvant chemotherapy	ctDNA, urinary EV-DNA, urinary ctDNA	Clinical performance reported; assessment phase NR.<br>Sensitivity/detection: Mutation detection sensitivity: plasma ctDNA 39.2% urinary EV-DNA 34.0% urinary ctDNA 21.6%	NR	NR	NR	NR	NR	NR	urinary extracellular vesicle DNA liquid biopsy analyzed using ddPCR
Kasi P. et al, 2022 [104]	10.7759/cureus.26648	Case report - descriptive evidence only	Age/sex: 43 year old woman<br>Region: United States & Pakistan<br>Cohort: 1	Systemic treatment: Larotrectinib, Pembrolizumab, Ipilimumab, Nivolumab	MLH1, PMS2, MSH2, MSH6, TPR-NTRK1 TRK RAS/RAF WT	Not assessed	Not assessed	Not assessed	Not assessed	Descriptive longitudinal monitoring; no comparative performance estimate.	NR	NR	Next-generation sequencing (NGS) and immunohistochemistry (IHC)
Kataoka K. et al, 2024 ESMO [32]	10.1016/j.annonc.2024.08.2240	GALAXY subgroup analysis; overlapping CIRCULATE-Japan cohort	Age/sex: 34 - 85<br>Region: Japan<br>Cohort: 190	Adjuvant treatment: adjuvant chemotherapy (ACT) with drugs such as 5-fluorouracil (5-FU) and oxaliplatin	ctDNA RAS BRAF MSI	NR	NR	NR	NR	NR	NR	NR	personalized, tumor-informed 16-plex polymerase chain reaction-next-generation sequencing (mPCR-NGS) assay Signatera
Kim, J. et al, 2025 [57]	10.3390/cancers17152560	Clinical cohort; prospective/retrospective design NR	Age/sex: ctDNA positive mean = 60 ctDNA negative mean = 66<br>Region: United States<br>Cohort: 28 patients locally advanced rectal cancer (LARC) 9 ctDNA	Perioperative treatment: total neoadjuvant therapy (TNT)	ctDNA	Analytical performance (not a clinical MRD phase).<br>Sensitivity/detection: 95% sensitivity (analytical, assay)<br>Specificity: 99.7% specificity (analytical, assay)	NR	Not assessed	Not assessed	Not assessed	NR	NR	Tumor-informed ctDNA assay (Signatera), next-generation sequencing (NGS) to infer up to 16

			positive 19 ctDNA. negative										patient- and tumor-specific somatic single-nucleotide variants in plasma cell-free DNA using a multiplex PCR-based approach
Kotani D. et al, 2023 [28]	10.1038/s41591-022-02115-4	GALAXY observational cohort; overlapping CIRCULATE-Japan population	Age/sex: ctDNA negative 25-93 (median =69) ctDNA positive 39-88 (median = 67)<br>Region: Japan<br>Cohort: 1,039 stage II-IV resectable CRC	Adjuvant treatment: adjuvant chemotherapy (ACT), capecitabine or capecitabine in combination with oxaliplatin-based therapy	ctDNA (up to 16 patient-specific clonal, somatic single-nucleotide variants (SNVs)	Analytical performance: >95% sensitivity at 0.01% mean VAF; 99.7% analytical specificity.	NR	MRD window at 4 weeks after surgery; formal clinical sensitivity/specificity NR.	Not assessed	Not assessed	Postsurgical ctDNA positivity: recurrence HR 10.0 (95% CI NR); stage II-III subgroup HR 10.82 (95% CI NR).	Observational GALAXY analysis suggested that MRD-positive stage II-III patients derived benefit from ACT; treatment-selection bias precludes causal inference.	whole-exome sequencing (WES) of formalin-fixed, paraffin-embedded (FFPE) tumor tissue followed by personalized multiplex polymerase chain reaction-based next-generation sequencing (NGS) (Signatera, Natera) of plasma cell free DNA (cfDNA).
Lee, J. Y. et al, 2026 [85]	10.1038/s41698-026-01452-8	Diagnostic/assay evaluation cohort	Age/sex: malignant group: 34-80 (mean = 61.1 years) benign group: 52-75 (mean = 63.4 years)<br>Region: Korea<br>Cohort: 58, 39 patients stage 0-IV and 19 benign patients	NR	KRAS mutations (codon 12, 13, 61, and 146)	NR	NR	NR	NR	NR	NR	NR	PNAClamp™ KRAS Mutation Detection Kit, KRAS mutation-positive were further analyzed by Sanger sequencing
Leon Arellan	10.3390/diagnostics12040993	Prospective postoperative	Age/sex: 20-80<br>Region: NR<br>Cohort: 28 CRC patients	usual CRC treatment	methylation of Septin 9 gene in circulating plasma	NR	Preoperative detection of CRC:	Recurrence detection: day 5-7 sensitivity	Not assessed	CEA at 6 months: sensitivity	NR	NR	SEPT9 gene methylation assay

o, M. et al, 2022 [38]		follow-up cohort			DNA CEA (6 month post surgery)		sensitivity 55.6%; specificity 100%.	100%, specificity 75%; 1 month sensitivity 100%, specificity 94.7%; 3 months sensitivity 66.7%, specificity 80%.		33%; specificity 86.4%.			
Li, X. et al, 2024 [45]	10.1038/s44321-024-00115-0	Assay development/validation cohort	Age/sex: NR<br>Region: China<br>Cohort: 32 CRC patients	NR	circulating variant allele frequencies (cVAF) of tumor-specific variants in cfDNA	Analytical VAF range reported: 0.4%-0.6%.	NR	Landmark within 6 weeks after surgery: sensitivity 90%; specificity 100%.	Not assessed	Not assessed	NR	NR	AccuScan uses whole genome sequencing (WGS) with single-read error correction for circulating tumor DNA (ctDNA) analysis.
Lin, Z. et al, 2025 [107]	10.1016/j.canlet.2025.217442	Neoadjuvant clinical cohort	Age/sex: 28-80 (median = 58)<br>Region: China<br>Cohort: 79 locally advanced rectal cancer (LARC) patients	Perioperative treatment: neoadjuvant therapy (NAT), sequential camrelizumab and chemotherapy following shortRT (SCRTc).	ctDNA, Carcinoembryonic antigen (CEA), Carbohydrate antigen 19-9 (CA19-9)	NR	NR	NR	NR	NR	NR	NR	Targeted deep sequencing using novel MRD panel
Liu W. et al, 2022 [58]	10.1016/j.ebiom.2022.103945	Clinical cohort	Age/sex: 29 - 68<br>Region: China<br>Cohort: 60 with locally advanced rectal cancer (LARC)	Perioperative treatment: neoadjuvant chemotherapy with capecitabine and oxaliplatin	KRAS, TP53, APC & SMAD4	Not assessed	Not assessed	Not assessed	Post-neoadjuvant treatment recurrence prediction: personalized assay sensitivity 76.47%, specificity 97.67%; universal panel sensitivity 66.7%, specificity	Not assessed	NR	NR	1.Personalized Assay targeting tumor-informed mutations 2.Universal panel of genes frequently mutated in CRC 3.Low depth sequencing for copy-number alterations (CNA)

									78.38%; copy-number approach sensitivity 66.7%, specificity 100%.				
Loeffler, C. et al, 2025 [59]	10.1038/s41467-025-62910-8	DACHS training cohort with GALAXY external validation; overlapping GALAXY population	Age/sex: DACHS cohort: >70 years =804 GALAXY cohort: >70 years = 604 Region: Germany and Japan Cohort: 1766 patients (DACHS training cohort) 1404 patients (GALAXY external validation cohort) stage II-IV colorectal cancer	Adjuvant treatment: adjuvant chemotherapy (ACT)	ctDNA	NR	NR	NR	NR	NR	NR	NR	transformer-based deep learning model analysis on hematoxylin & eosin stained whole slide images. ctDNA detection method based on a tumor informed assay (Signatera, Natera Inc.) Tumor specific somatic single-nucleotide variants (SNVs) were identified via whole-exome sequencing (WES).
Lonardi S. et al, 2022 [24]	10.3390/ijms231911529	Exploratory analysis - PREDATOR study	Age/sex: median - 60.1 Region: Italy & United States Cohort: 69	NR	KRAS, NRAS, APC, TP53, PIK3CA, BRCA1, EGFR, ROS1, KMT2D, POLE, and ABL1	Analytical performance (not a clinical MRD phase). Sensitivity/detection: overall sensitivity at the follow-up time point was 69% VAF threshold from 0.014-56.5% Specificity: overall specificity at the follow-up time point was 100%	NR	Not assessed	Not assessed	Follow-up ctDNA performance: sensitivity 69%; specificity 100%. Analytical VAF range 0.014%-56.5%.	MRD-positive vs negative: DFS HR 4.97 (95% CI 2.67-9.24); OS HR 27.05 (95% CI 3.60-203.46).	NR	tissue comprehensive genomic profiling (CGP)-informed ctDNA assay: “FoundationOne Tracker”
Loupakis F. et al, 2021	10.1200/PO.21.00101	Clinical cohort; prospective/retr	Age/sex: median - 60.1 Region: Italy Cohort: 112	Adjuvant treatment: Cancer treatments in compliance with	KRAS mutations: G12A, G12C, G12D, G12R,	Not assessed	Not assessed	Postoperative personalized mPCR-NGS	Not assessed	Not assessed	Postoperative MRD was	NR	personalized and tumor-informed

[60]		ospective design NR		regional standard of care regimens ACT	G12S, G12V, and G13D			sensitivity 84.6%; ddPCR sensitivity 38.4%; reported specificity 93.3%.			associated with recurrence after metastasectomy; exact HR/CI NR in the extraction table.		ctDNA assay (bespoke multiple PCR, next-generation sequencing assay) WGS; selected 16 patient-specific somatic clonal variants ddPCR analysis
Lu, Y. et al, 2026 [39]	10.1002/jso.70294	Single-institution observational cohort	Age/sex: mean = 50.8<br>Region: China<br>Cohort: 100 locally advanced rectal cancer (LARC) or locally recurrent rectal cancer (LRRC) patients	Systemic treatment: FOLFOX (oxaliplatin, fluorouracil, and leucovorin) or CAPEOX (capecitabine and oxaliplatin) chemotherapy	ctDNA, CEA (comparative biomarker)	Analytical VAF threshold: 0.004%.	Preoperative positivity/detection (not MRD sensitivity): ctDNA 100%; CEA 47%.	Postoperative recurrence/MRD detection: ctDNA sensitivity 78.6%, specificity 81.9%; CEA sensitivity 21.4%, specificity 79.2%.	Post-adjuvant therapy: ctDNA sensitivity 96.4%, specificity 80.6%; CEA sensitivity 42.9%, specificity 72.2%.	Not assessed	NR	NR	Tumor-informed ctDNA analysis (brPROPHET platform), whole-exome sequencing (WES) and personalized mutation panel
Luo J. et al, 2023 [105]	10.1097/MD.0000000000034330	Case report - descriptive evidence only	Age/sex: 45 year old man<br>Region: China<br>Cohort: 1	Systemic treatment: The patient received systemic therapy and local treatment, including FOLFOX, XELOX, and a combination therapy of bevacizumab plus FOLFIRI	KRAS (particularly the G12V mutation), APC, TP53, TCF7L2	Not assessed	Not assessed	Not assessed	Not assessed	Descriptive longitudinal monitoring; no comparative performance estimate.	NR	NR	next-generation sequencing-based ctDNA profiling tracking ultra-low-frequency somatic tumor mutations
Lutfi A. et al, 2023 CUREUS [61]	10.7759/cureus.43391	Case report - descriptive evidence only	Age/sex: 50 year old male<br>Region: United States<br>Cohort: 1	Systemic treatment: Pembrolizumab (PD-1 blockade) and FOLFOXIRI with bevacizumab	ctDNA & POLE	Not assessed	Not assessed	Not assessed	Not assessed	Descriptive longitudinal monitoring; no comparative performance estimate.	NR	NR	Next-generation sequencing (NGS) and ctDNA assay using tumor-informed MRD ctDNA SignateraTM assay

Lutfi A. et al, 2023 KARGER [78]	10.1159/000535174	Case report - descriptive evidence only	Age/sex: 62 year old male Region: United States Cohort: 1 Stage III CRC	Glecaprevir/pibrentasvir, a combination of HCV NS3/4A protease inhibitor and an HCV NS5A inhibitor	ctDNA RAS/RAF	Not assessed	Not assessed	Not assessed	Not assessed	Descriptive longitudinal monitoring; no comparative performance estimate.	NR	NR	Liquid biopsy assays False positive plasma only assay- false signaling due to active hepatitis C
Mallampati, S. [106]	10.1016/j.jmoldx.2019.06.008	Assay development/validation cohort	Age/sex: mean = 55.3 Region: United States Cohort: 32 stage IV colorectal adenocarcinoma patients	NR	TP53, APC, KRAS, NRAS, BRAF, PIK3CA, and ERBB2 (plus hotspot coding exons from 16 other genes)	Clinical performance reported; assessment phase NR. Sensitivity/detection: 87.23% (95% CI, 74.26%-95.17%) Specificity: 96.91% (95% CI, 95.11%-98.19%)	NR	NR	NR	NR	NR	NR	CRC23 assay, duplex sequencing of cfDNA
Martín-Arana, J. et al, 2025 [79]	10.1038/s43018-025-00960-z	Discovery and external validation cohorts	Age/sex: discovery cohort: median = 74 validation cohort: median = 64 Region: Spain (Discovery cohort), Denmark (Validation cohort) Cohort: discovery cohort: 25 validation cohort: 15	Adjuvant treatment: discovery cohort: adjuvant chemotherapy (ACT) with either capecitabine or capecitabine + oxaliplatin (CAPOX) validation cohort: adjuvant chemotherapy (ACT) with treatment regimens including, CAPOX, fluorouracil + oxaliplatin, folinic acid + fluorouracil + oxaliplatin, capecitabine or intravenous fluorouracil	ctDNA	Clinical performance reported; assessment phase NR. Sensitivity/detection: 89% (discovery), 100% (validation) (WES-TA approach; participant with positive ctDNA defined by the detection of only one mutation in plasma rather than two) Specificity: 95%	NR	NR	NR	NR	NR	NR	whole exome sequencing on longitudinal plasma samples followed by WES tumor-agnostic (WES-TA) ctDNA analysis
Miyazaki, K. et al, 2026 [103]	10.1186/s12943-026-02624-7	Training and testing cohorts	Age/sex: training cohort: 33-95 (median = 74) testing cohort: 35 - 87 (median = 65) Region: Spain (training), Japan (testing) Cohort: 175 stage II-III CRC patients	Perioperative treatment: surgery, neoadjuvant therapy, Adjuvant chemotherapy (ACT)	miRNA	Clinical performance reported; assessment phase NR. Sensitivity/detection: 86% Specificity: 77%	NR	NR	NR	NR	NR	NR	exosome RNA isolation, RT-qPCR

Mo, S. et al, 2023 [41]	10.1001/jamaoncol.2023.0425	Clinical cohort	Age/sex: mean (SD) = 60.1 (10.3)<br>Region: China<br>Cohort: 299 patients with stage I to III CRC	Adjuvant treatment: Adjuvant chemotherapy (ACT)	ctDNA methylation (6 markers)	Not assessed	Not assessed	Postoperative methylation markers: individual-marker sensitivity 26%-52%; specificity 95%-98%.	Not assessed	Not assessed	Methylated ctDNA was an independent recurrence predictor as early as 1 month after surgery; combination with CEA improved prognostic performance. Exact HR/CI NR in the extraction table.	NR	multiplex quantitative polymerase chain reaction (qPCR) for plasma ctDNA methylation analysis
Mögele, T. et al, 2024 [96]	10.3390/cancers16244173	NEORECT trial cohort	Age/sex: 37-87 (median = 68)<br>Region: Germany<br>Cohort: 40 stage II-III rectal cancer patients (26 patients with complete dataset)	Perioperative treatment: neoadjuvant chemoradiotherapy	ctDNA	NR	NR	NR	NR	NR	NR	NR	next generation sequencing (NGS), digital PCR
Molinari, C. et al, 2024 [62]	10.1038/s41598-024-80855-8	Clinical cohort; prospective/retrospective design NR	Age/sex: 63-76 (median = 67)<br>Region: Italy<br>Cohort: 30 stage II-III locally advanced rectal cancer (LARC) patients	Perioperative treatment: neoadjuvant chemoradiotherapy followed by surgery	ctDNA	NR	NR	NR	NR	NR	NR	NR	tumor-informed, 16-plex PCR assay (Signatera™, Natera, Inc.) whole-exome sequencing (WES) patient-specific assay (targeting 16 somatic single-nucleotide variants) using an mPCR-NGS approach
Nakamura Y. et	10.1158/1078-	Clinical cohort; prospective/retr	Age/sex: 20 years and older	Adjuvant treatment: ACT	ctDNA	Not assessed	Not assessed	Posttreatment landmark	Posttreatment assessment	Longitudinal sensitivity:	Median molecular	NR	Guardant Reveal assay,

al, 2024 AACR [80]	0432.CCR-24-1651	ospective design NR	Region: Japan<br>Cohort: 342					performance was evaluated; exact landmark sensitivity NR in the extraction table. Specificity across posttreatment samples was 98.2%.	after definitive therapy; values are reported under landmark/longitudinal findings.	81% for stage II or higher colon cancer and 60% for rectal cancer. Sensitivity by recurrence site: liver 100%, lung 53%, peritoneum 40%.	lead time 5.3 months; recurrence-free interval differed according to ctDNA status. Exact HR/CI NR in the extraction table.		which involves next-generation sequencing and bioinformatic analysis
Nakamura Y. et al, 2024 Nature [26]	10.1038/s41591-024-03254-6	GALAXY cohort analysis; overlapping CIRCULATE-Japan population	Age/sex: 28 - 95 (median - 69)<br>Region: Japan<br>Cohort: 2240 2109 included in analysis<br>Stage II, III, IV CRC	Adjuvant treatment: ACT	Wildtype RAS/BRAF, MSI, BRAF V600E KRAS G12C, ERBB2 amplification TP53 Y220C, NTRK1/2/3 fusion RET fusion	Analytical assay: >95% sensitivity at 0.01% mean VAF; 99.7% analytical specificity.	NR	MRD-window ctDNA status; clinical sensitivity/specificity NR in this analysis.	During ACT, sustained versus transient ctDNA clearance was evaluated.	Serial surveillance; performance metrics NR in this analysis.	MRD-positive vs negative: DFS HR 11.99 (95% CI NR); OS HR 9.68 (95% CI NR). Sustained vs transient clearance: 24-month DFS 89.0% vs 3.3%; 24-month OS 100.0% vs 82.3%.	Observational findings support risk stratification and suggest ACT benefit in MRD-positive patients; not randomized evidence.	Tumor-informed ctDNA testing was performed using a personalized, 16-plex polymerase chain reaction (PCR) next-generation sequencing (NGS) assay (Signatera, Natera, Inc.)
Nakamura Y. et al, 2025 Text [16]	10.1158/1078-0432.CCR-24-2396	GALAXY subset analysis; overlapping CIRCULATE-Japan population	Age/sex: 37 - 89 (median - 67.7)<br>Region: Japan & Taiwan<br>Cohort: 80; 69 evaluable patients	Adjuvant treatment: ACT	ctDNA-MRD status itself	Not assessed	Not assessed	Landmark at 4 weeks: sensitivity 61.1%; specificity 87.9%.	Not assessed	Longitudinal assessment: sensitivity 83.3%; specificity 89.5%.	ctDNA positivity predicted recurrence; exact HR/CI NR in the extraction table.	NR	tumor-naive ctDNA assay integrates methylation and genomic variant data Illumina NovaSeq
Nakamura, Y. et al, 2026	10.1038/s41698-026-01277-5	GALAXY assay validation cohort; overlapping	Age/sex: 29-88 (median = 68)<br>Region: Japan	Adjuvant treatment: Adjuvant chemotherapy (ACT)	ctDNA differentially methylated regions (DMRs)	Not assessed	Not assessed	MRD window: sensitivity 58.5%; specificity	Not assessed	Longitudinal sensitivity 84.4%; specificity	ctDNA positivity: DFS HR 10.0 in the	Observational high-risk stage II-III subgroup	methylation-based, tissue-free assay

[43]		CIRCULATE-Japan population	Cohort: 195 patients with stage I-IV CRC					100% in patients who did not receive ACT.		92.1% at patient level and 97.2% at sample level; median lead time 4.6 months.	MRD window and HR 31.9 during surveillance (95% CIs NR).	suggested ACT benefit in ctDNA-positive patients (adjusted HR 0.014); requires prospective validation.	(LatitudeTM assay)
Nakano T. et al, 2023 [97]	10.1111/cas.16149	Assay development/validation cohort	Age/sex: NR<br>Region: Japan<br>Cohort: 173 stage III CRC	Adjuvant treatment: Capecitabine plus oxaliplatin (CAPOX) as adjuvant chemoradiotherapy (ACT)	FGD5, GPC6 & MSC	Clinical performance reported; assessment phase NR. Sensitivity/detection: sensitivity of the AMUSE assay was 78.6% Specificity: specificity of the AMUSE assay was 89.5%	NR	NR	NR	NR	NR	NR	Digital polymerase chain reaction (dPCR) using the AMUSE assay
Nguyen H. et al, 2022 [13]	10.3389/fonc.2022.1069296	Clinical cohort; prospective/retrospective design NR	Age/sex: 27 - 85 (median - 60)<br>Region: Vietnam<br>Cohort: 103 Stage I-IV CRC	Systemic treatment: Cetuximab and Panitumumab	APC, TP53, KRAS (G12 D/S/V/C/A), RNF213, BRAFV600E, ATR, TCF7L2, AMERI, RNF43Additionally, genes such as FAT4, NRAS, PIK3CA, and TRRAP are highlighted	Analytical VAF threshold: 0.05%-10%; assay specificity >99%.	Preoperative ctDNA detection rate 90.5% (positivity, not MRD sensitivity).	Not assessed	Not assessed	Not assessed	NR	NR	Qiagen personalized multiplex PCR and ultra-deep sequencing NEBNext Ultra II FS-library prep kit with panel of 95 targeted genes K-TRACK
Nguyen, H. et al, 2024 [63]	10.1016/j.esmorw.2024.100076	Clinical trial and real-world cohort	Age/sex: 27-85 (median = 60)<br>Region: Vietnam<br>Cohort: 92 stage I-IVA CRC patients	Adjuvant treatment: surgery ± adjuvant chemotherapy	ctDNA	NR	Preoperative ctDNA detection rate 91.2% (positivity, not MRD sensitivity); CEA elevation 42.9%.	At 2-4 weeks after surgery: clinical sensitivity/specificity NR.	NR	Longitudinal recurrence detection: sensitivity 100%; specificity 100%; median lead time 8.0 months.	Postoperative DFS HR 30.3 (95% CI 3.3-278.9). Longitudinal positivity HR 173.6 (95% CI 19.7 to >1000); real-world postoperative DFS	NR	personalized tumor-informed ctDNA assay, MRD testing using the K-Track™ assay, mPCR to detect ctDNA in the plasma

											HR 44.2 (95% CI 9.2-212.0).		
Nguyen, T. et al, 2025 [89]	10.1177/17588359251393090	Multimodal assay development cohort	Age/sex: NR Region: Vietnam Cohort: 51 colorectal cancer patients	NR	ctDNA	Clinical performance reported; assessment phase NR. Sensitivity/detection: 80% Mutations detected solely by mPCR VAF: (median 0.24%) VAF of cfDNA samples: 0.11%-11.13% Specificity: 100%	NR	NR	NR	NR	NR	NR	Multiplex PCR (mPCR), shallow whole-genome sequencing (sWGS), amplicon and hybridization sequencing, copy number alteration (CNA) and fragmentomics of cfDNA
Ohta R. et al, 2021 [98]	10.1016/j.ejso.2021.07.017	Clinical cohort; prospective/retrospective design NR	Age/sex: over 20 years Region: Japan Cohort: 200	NR	KRAS mutation sites in exon-2: G12A, G12C, G12D, G12R, G12S, G12V, and G13D	Clinical performance reported; assessment phase NR. Sensitivity/detection: sensitivity of detection using ctDNA was 29.8%, and using trtDNA was 33.3% ctDNA+trtDNA: 53.6% Specificity: specificity of detection using ctDNA was 94.8%, and using trtDNA was 93.1%	NR	NR	NR	NR	NR	NR	Droplet digital PCR (ddPCR) was used to detect KRAS mutations
Pan, S., & Wang, G., 2026 [64]	10.1016/j.suronc.2026.102492	Clinical cohort; prospective/retrospective design NR	Age/sex: 63.8 ± 10.7 (mean ± SD) Region: China Cohort: 1200 patients with stage II-III colorectal cancer	Adjuvant treatment: adjuvant chemotherapy (ACT)	ctDNA	NR	NR	NR	NR	NR	NR	NR	tumor-informed personalized ctDNA assay
Pan, S., & Wang, G., 2026 [75]	10.1002/wjs.70360	Clinical cohort; prospective/retrospective design NR	Age/sex: 64.7 ± 9.8 (mean ± SD) Region: China Cohort: 950 patients with stage II-III colorectal adenocarcinoma	Perioperative treatment: neoadjuvant therapy	Aging Shock Index (ASI, systemic stress phenotype, derived from inflammatory, immune surveillance, and immunometabolis	NR	NR	NR	NR	NR	NR	NR	tumor-informed targeted next-generation sequencing assay

					m biomarkers), ctDNA-defined MRD								
Pan, S., & Wang, G., 2026 [65]	10.1007/s00464-026-12915-1	Clinical cohort; prospective/retrospective design NR	Age/sex: 62.1±8.7 (mean ± SD)<br>Region: China<br>Cohort: 1000 stage II-III colorectal adenocarcinoma patients	Adjuvant treatment: adjuvant chemotherapy (ACT)	ctDNA-MRD	Clinical performance reported; assessment phase NR.<br>Sensitivity/detection: NR; VAF detection range: 10^-4 to 10^-5	NR	NR	NR	NR	NR	NR	tumor-informed ctDNA assay
Parikh A. et al, 2021 Oct [84]	10.1158/1078-0432.CCR-21-0410	Prospective clinical cohort	Age/sex: 35 - 84 (median - 60)<br>Region: United States<br>Cohort: 103	NR	ctDNA	Not assessed	Not assessed	Not assessed	Landmark approximately 1 month after definitive therapy: sensitivity 55.6%; specificity 100%; PPV 100%.	Sensitivity improved to 69% with longitudinal samples and 91% with surveillance samples near recurrence.	Landmark ctDNA positivity: recurrence HR 11.28 (95% CI NR). CEA did not significantly predict recurrence (HR 1.84, P=0.18).	NR	plasma-only ctDNA assay integrating genomic and epigenomic cancer signatures
Parikh A. et al, 2024 [23]	10.1158/1078-0432.CCR-23-3660	Prospective clinical cohort	Age/sex: 39 - 75 (median - 56)<br>Region: United States<br>Cohort: 52	Local/curative-intent treatment: Curative intent procedures	ctDNA (ctDNA detected/not detected)	NR	Preprocedure ctDNA status assessed; not interpreted as postoperative MRD sensitivity.	Primary postprocedure timepoint (~3 weeks): sensitivity 58.1% for any future recurrence and 77.5% for recurrence within 1 year; specificity 90.9%.	Not assessed	Longitudinal sampling improved recurrence detection; exact performance values are presented in the source article.	Postprocedure ctDNA positivity: RFS HR 5.27 (95% CI 2.31-12.0); OS HR 12.83 (95% CI 3.6-45.9).	NR	the Guardant Reveal assay Tumor naive
Pellatt, A. J. et al, 2025 [66]	10.1200/po-25-00142	Phase II trial - INTERCEPT-TT	Age/sex: 37-73 (median = 60.2)<br>Region: United States<br>Cohort: 15 stage II to IV CRC patients	Adjuvant treatment: Adjuvant chemotherapy (ACT), tipiracil combined with trifluridine	ctDNA	NR	NR	NR	All participants had ctDNA-defined MRD after prior adjuvant therapy; post-adjuvant baseline before TAS-102.	ctDNA assessed every 3 months with imaging.	Median DFS 9.4 months with TAS-102 vs 5.75 months in synthetic controls (P=0.03).	INTERCEPT-TT: ctDNA clearance at 3 months 47% and at 6 months 36%; synthetic-control spontaneous clearance 6.7% at 3	tumor-informed ctDNA assay (Signatera)

												months. Clearance was often transient and did not establish long-term cure.	
Pellini B. et al, 2021 [33]	10.1200/PO.20.00276	Clinical cohort; prospective/retrospective design NR	Age/sex: median - 57<br>Region: United States<br>Cohort: 24 patients with oligometastatic CRC	Perioperative treatment: neoadjuvant chemotherapy	TP53, APC, KRAS, PIK3CA, FBXW7, SOX9, and NRAS	Analytical performance: VAF threshold 0.02%-38.31%; plasma ctDNA MRD detection sensitivity 95% and specificity 100% in the full cohort.	NR	Not assessed	After neoadjuvant treatment: sensitivity 94% among treated patients; specificity 100%.	Not assessed	NR	NR	AVENIO targeted next-generation sequencing. Tumor naive with 197 genes that are frequently mutated being assessed
Qi Z. et al, 2023 [12]	10.1002/cam4.6286	Clinical cohort; prospective/retrospective design NR	Age/sex: 29 -79 (median - 60)<br>Region: China<br>Cohort: 127	NR	ctDNA	Clinical performance reported; assessment phase NR. Sensitivity/detection: detection of three positive sites was sufficient to achieve nearly 100% overall sample level sensitivity Ranges from 93.0-100% depending on SNV threshold Specificity: specificity of the ctDNA MRD detection was 98.9% Ranges from 93.1-100% depending on SNV threshold	NR	NR	NR	NR	NR	NR	Qiagen, Illumina OriMIRACLES ultradeep sequencing of plasma-free DNA using a tumor-informed PCR based NGS assay WES
Reinert T. et al, 2022 [36]	10.1002/ijc.33924	Prospective clinical cohort	Age/sex: 45.1 - 89.4 (median - 67.7)<br>Region: Denmark<br>Cohort: 68	Adjuvant treatment: Surgery, ACT	APC, BRAF, KRAS, NRAS, PIK3CA & TP53	Not assessed	Not assessed	Not assessed	Not assessed	Surveillance recurrence detection: sensitivity 72.3%; specificity 100%.	Postoperative and serial ctDNA were associated with recurrence; exact HR/CI NR	NR	Qiagen QX200 ddPCR system ddPCR KRAS G12/G13 screening kit MSK-IMPACT panel- covers 468 genes

											in the extraction table.		
Rubio-Alarcón, C. et al, 2025 [37]	10.1093/bjs/znaf281	Multicentre prospective cohort - PROVENC3	Age/sex: ctDNA positive: 43-83 (median = 68) ctDNA negative: 32-79 (median = 63) Region: Netherlands Cohort: 209 stage III colon cancer	Adjuvant treatment: adjuvant chemotherapy (ACT) (209 patients), capecitabine + oxaliplatin (CAPOX) (188 of 209 patients), others were treated with 5-fluorouracil + leucovorin + oxaliplatin (FOLFOX) or capecitabine monotherapy	ctDNA	Not assessed	Not assessed	Not assessed	Not assessed	Surveillance sensitivity 77% (10/13); median lead time 7.6 months before clinical recurrence. Analytical VAF threshold 0.035%.	NR	NR	presence of minimal residual disease was determined using Labcorp® Plasma Detect™, a novel tumour-informed whole genome sequencing (WGS) ctDNA test
Ryoo S. et al, 2023 [11]	10.1038/s41416-023-02300-3	Clinical cohort; prospective/retrospective design NR	Age/sex: median - 67 Region: Korea Cohort: 98 stage II, III CRC	Adjuvant treatment: Surgery, ACT	Personalized SNV to detect ctDNA and MRD	Not assessed	Not assessed	Postoperative 3-week MRD positivity predicting 3-year recurrence: sensitivity 61.9%; specificity 83.9%.	Not assessed	Not assessed	NR	NR	Individual variants identified in tumor WES Alpha-Liquid Detect- a tumor-informed personalized MRD detection assay Illumina to identify genetic mutations
Salimiaghdam, N. et al, 2026 [67]	10.21873/cdp.10578	Case series - descriptive evidence only	Age/sex: 53-66 Region: United States Cohort: 4 stage III CRC	Adjuvant treatment: adjuvant chemotherapy (ACT)	ctDNA	Not assessed	Not assessed	Not assessed	Not assessed	Descriptive longitudinal monitoring; no comparative performance estimate.	NR	NR	tumor-informed ctDNA assay (Signatera), multiplex polymerase chain reaction (PCR), next generation sequencing (NGS)
Schøler L. et al, 2017	10.1158/1078-	Clinical cohort; prospective/retrospective design NR	Age/sex: NR Region: Denmark Cohort: 45	Local/curative-intent treatment: Chemotherapy, radiation therapy,	ctDNA somatic structural variants (SSV) and somatic point mutations	Not assessed	Not assessed	Postsurgical relapse detection: sensitivity	Not assessed	Serial monitoring preceded clinical	All ctDNA-positive patients	NR	Qiagen Tumor-informed ddPCR for

[15]	0432.CCR-17-0510			radiofrequency ablation, surgery	(SPM) KRAS: Specific focus on mutations in codons G12V/A/C/D/S, G13D, and Q61H. BRAF: BRAF V600E			100%; specificity 100%.		recurrence; exact lead time varies across patients.	relapsed during follow-up, whereas no ctDNA-negative patient relapsed in the reported cohort.		ctDNA quantification WES
Sinicrope, F. et al, 2026 [82]	10.1200/JCO-25-02086	Phase III trial secondary analysis - Alliance N0147	Age/sex: 19.4-87.0 (median = 58.5) Region: United States Cohort: 2260 (available for ctDNA analysis), stage III colon cancer ctDNA detected = 461 ctDNA not detected = 1799	Systemic treatment: FOLFOX alone or combined with cetuximab	epigenomic ctDNA methylation patterns	Analytical specificity >98% for the tissue-free epigenomic assay.	NR	Plasma collected after surgery and before adjuvant FOLFOX; ctDNA-positive 20.4%. Clinical sensitivity/specificity for recurrence NR.	Not assessed	Not assessed	ctDNA-positive vs negative: TTR HR 5.96 (95% CI 5.11-6.96); DFS HR 5.03 (95% CI 4.36-5.81); OS HR 4.45 (95% CI 3.76-5.27). Five-year DFS 27.7% vs 77.1%.	NR	cell-free DNA extracted from plasma, a tissue-free epigenomic assay (Guardant Reveal), ctDNA positive cases were evaluated with the Guardant360 assay
Siravegna G. et al, 2017 [100]	10.1093/annonc/mdx095	Case report - descriptive evidence only	Age/sex: 53 year old Region: Italy Cohort: 1	Systemic treatment: CT monitoring, chemotherapy, oxaliplatin, 5FU, Leucovorin, Bevacizumab,Entrectinib	ctDNA: CAD-ALK gene rearrangement, TP53 mutation & Emerging ALK kinase mutations	Not assessed	Not assessed	Not assessed	Not assessed	Descriptive longitudinal monitoring; no comparative performance estimate.	NR	NR	Qiagen for extraction and purification Droplet digital PCR (ddPCR) and end-point PCR Analyzed with QX200 droplet reader NGS to profile ctDNA
Slater S. et al, 2024 [83]	10.1158/1078-0432.CCR-24-0226	Prospective study - TRACC Part B	Age/sex: ≥18 years Region: United Kingdom Cohort: 1,203 patients were enrolled, and plasma samples from 214 patients were	NR	Tissue-Free Samples: ctDNA detected/not detected Tissue Assay: 84 genes analyzed. Most	Not assessed	Not assessed	Landmark sensitivity 43.8%; specificity 93.0%. Postoperative ctDNA-	Not assessed	Longitudinal sensitivity 62.1% (95% CI 42.2%-79.3%); specificity 85.9% (95%	NR	NR	The Guardant Reveal assay, which evaluates genomic and methylation signals using a

			analyzed with a new histologic diagnosis of stage I to III colorectal cancer (CRC)		common: RAS, RAF, MSI			positive/confirmed recurrence analysis: sensitivity 41.7%; specificity 90.8%.		CI 78.9%-91.3%).			multiomic next-generation sequencing (NGS) platform
Sørensen, E. et al, 2026 [68]	10.1186/s12967-026-07762-6	Assay validation cohort	Age/sex: NR Region: Denmark Cohort: 144 patients with stage III colorectal cancer	Adjuvant treatment: adjuvant chemotherapy (ACT)	Somatic structural variants (SVs) detected in ctDNA	Clinical performance reported; assessment phase NR. Specificity: 99%	NR	NR	NR	NR	NR	NR	tumour informed whole-exome sequencing based ctDNA detection using somatic structural variants (SVs) ddPCR analysis based on whole-exome sequencing, deep-targeted sequencing based on whole-exome sequencing
Tarazona N. et al, 2020 [101]	10.1136/esmoopen-2020-000847	Clinical cohort; prospective/retrospective design NR	Age/sex: NR Region: Spain Cohort: 150 stage I-IV CRC	NR	CDX2 expression and circulating tumor DNA (ctDNA) CDX2 IL-6	NR	NR	NR	NR	NR	NR	NR	Qiagen for extraction and purification ctDNA was tracked using droplet digital PCR, and CDX2 expression was analyzed by immunostaining ELISA to evaluate cytokines
Tarazona, N. et al, 2019 [102]	10.1093/annonc/mdz390	Prospective clinical cohort	Age/sex: NR Region: Spain Cohort: 150 localized colon cancer patients	Adjuvant treatment: adjuvant chemotherapy	ctDNA	Not assessed	Not assessed	Postoperative ctDNA positivity assessed; formal	Post-adjuvant ctDNA positivity assessed; formal	Serial plasma assessment; median lead	Postoperative ctDNA positivity: DFS HR 17.56 (95%	NR	custom 29 gene panel targeted next-generation sequencing

			(94 patients collected samples)					sensitivity/specificity NR.	sensitivity/specificity NR.	time 11.5 months.	CI NR). Post-adjuvant ctDNA positivity: early-relapse HR 10.02 (95% CI NR). Serial plasma positivity: DFS HR 11.33 (95% CI NR).		(NGS), droplet digital PCR (ddPCR) ctDNA assay
Tie J et al, 2019 [20]	10.1001/jamaoncol.2019.3616	Prospective clinical cohort	Age/sex: 26-82 (median - 64)<br>Region: Australia<br>Cohort: 100 patients were initially recruited, with 96 eligible patients included in the analysis<br>Stage III CRC	Adjuvant treatment: Adjuvant chemotherapy, specifically oxaliplatin-based therapy and fluoropyrimidine	APC, TP53, KRAS, NRAS, PIK3CA, SMAD4, BRAF, FBXW7, TCF7L2, and PTEN	Not assessed	Not assessed	Postsurgical ctDNA detectable in 20/96 (21%).	Postchemotherapy ctDNA detectable in 15/88 (17%). Three-year RFI: 30% ctDNA-positive vs 77% ctDNA-negative.	Not assessed	Postsurgical recurrence HR 3.8 (95% CI NR); adjusted postsurgical RFI HR 7.5 (95% CI 3.5-16.1); postchemotherapy RFI HR 6.8 (95% CI NR). Serial postsurgical and postchemotherapy ctDNA strongly stratified recurrence risk; no ctDNA-guided intervention was tested.	NR	Massively parallel sequencing and personalized Safe-Sequencing System (Safe-SeqS) assays for personalized mutations indicating ctDNA

Tie J. et al, 2021 [69]	10.1371/journal.pmed.1003620	Prospective clinical cohort	Age/sex: 30.8 - 84.7 (median - 64)<br>Region: Australia<br>Cohort: 54	Adjuvant treatment: ACT	SMAD4, TP53, AKT1, APC, BRAF, CTNNB1, ERBB3, FBXW7, HRAS, KRAS, NRAS, PIK3CA, PPP2R1A, RNF43, POLE	NR	Preoperative ctDNA detection rate 85% (positivity, not MRD sensitivity).	Postoperative ctDNA detection rate 24%; clinical specificity NR.	Not assessed	Not assessed	Postoperative ctDNA dynamics were associated with recurrence risk after resection of colorectal liver metastases; exact HR/CI NR in the extraction table.	NR	Qiagen for extraction and purification Safe-SeqS assay (personalized assay) was used to detect ctDNA (somatic mutations)
Tie, J. et al, 2017 [22]	10.1126/scitranslmed.aaf6219	Prospective clinical cohort	Age/sex: 23-87 (median = 65)<br>Region: Australia<br>Cohort: 230 stage II colon cancer	Adjuvant treatment: 52 (23%) patients received adjuvant chemotherapy (ACT)	patient-specific somatic mutations	Not assessed	Not assessed	Postoperative recurrence prediction at 36 months: sensitivity 48%; specificity 98% (false-positive rate 2%).	After completion of chemotherapy, ctDNA status was assessed; formal sensitivity/specificity NR.	Not assessed	Without ACT, recurrence occurred in 79% of postoperative ctDNA-positive vs 9.8% of ctDNA-negative patients; HR 18 (95% CI 7.9-40). After completion of chemotherapy, ctDNA positivity was associated with inferior RFS: HR 11 (95% CI 1.8-68).	NR	massively parallel sequencing using Safe Sequencing System (Safe-SeqS) assays targeting patient specific ctDNA mutations

Wang Y. et al, 2021 [111]	10.1371/journal.pmed.1003741	Prospective clinical cohort	Age/sex: median - 57<br>Region: China<br>Cohort: 119 103 completed the study	Perioperative treatment: neoadjuvant chemoradiotherapy (nCRT) which included concurrent capecitabine and irinotecan chemotherapy	Most common:TP53, APC, KRAS. BRAF, PTEN, and PIK3CA MLH1, MLH3, MSH2, MSH6	Not assessed	Not assessed	Not assessed	Post-neoadjuvant/end-of-treatment NR	Not assessed	NR	NR	deep targeted panel sequencing of 422 cancer-related genes
Wang, P. et al, 2026 [40]	10.1080/07853890.2026.2694796	Clinical cohort; prospective/retrospective design NR	Age/sex: NR<br>Region: China<br>Cohort: 68 stage I-IV CRC patients	NR	methylation status of the SEPTIN9 gene (mS9) in circulating tumor DNA (ctDNA), Carcinoembryonic antigen (CEA) (comparative biomarker)	Not assessed	Not assessed	Combined methylated SEPTIN9 plus CEA at 1 year: sensitivity 86.7%; specificity 93.1%.	NR	NR	Postoperative methylated SEPTIN9 positivity was associated with increased recurrence risk; combined testing improved prognostic performance. Exact HR/CI NR in the extraction table.	NR	methylated SEPTIN9 (mS9) assay
Weinberg, B. A. et al, 2021 [70]	10.1159/000520743	Case report - descriptive evidence only	Age/sex: 35 year old<br>Region: United States<br>Cohort: 1 stage IIA colon cancer (colon adenocarcinoma) patient	NR	ctDNA	Not assessed	Not assessed	Not assessed	Not assessed	Descriptive longitudinal monitoring; no comparative performance estimate.	NR	NR	personalized NGS-mPCR ctDNA assay (Signatera)
Yang J. et al, 2024 [25]	10.4143/crt.2023.1371	Clinical cohort	Age/sex: 57 - 71 (median - 67)<br>Region: China<br>Cohort: 75 patients with resectable stage I-III colorectal cancer (CRC)	NR	TP53, APC, KRAS(G12D/V, G13D), PIK3CA, SMAD4, BRCA2, POLE, ERBB2, & PTEN	NR	Preoperative positivity (not MRD sensitivity): tumor-informed fixed assay 73.3%; tumor-agnostic fixed assay 57.3%.	Postoperative day 7 ctDNA status; formal sensitivity/specificity NR.	Not assessed	Not assessed	Postoperative ctDNA positivity: DFS HR 20.74 (95% CI 7.19-59.83); multivariable HR	NR	Qiagen for extraction and purification Whole-exome sequencing (WES) based tumor-informed assay and

											28.57 (95% CI 7.10-114.9). Preoperative ctDNA status did not predict DFS.		panel-based personalized assays ColonCore-fixed panel 41 genes OncoScreen Plus Panel WES via Twist Human Core Exome Kit
**Additional pivotal randomized evidence incorporated during peer review (not counted within the original 94-publication structured-search dataset)**													
Tie, J. et al., 2022 [29]	https://doi.org/10.1056/NEJMoa2200075	Randomized DYNAMIC trial (stage II colon cancer)	455 randomized patients with stage II colon cancer; Australia	ctDNA-guided versus standard clinicopathologic management; oxaliplatin-based or fluoropyrimidine ACT for ctDNA-positive patients	Tumor-informed personalized ctDNA assay	Not assessed	Not assessed	ctDNA assessed at 4 and/or 7 weeks after surgery. Three-year RFS: 86.4% in treated ctDNA-positive patients and 92.5% in untreated ctDNA-negative patients.	EOT ctDNA outcomes reported in the later 5-year analysis [30].	Follow-up for recurrence; phase-specific assay sensitivity/specificity NR.	Postoperative ctDNA classified recurrence risk; exact HR/CI NR in the original trial abstract.	ACT use decreased from 28% to 15%. Two-year RFS was noninferior: 93.5% ctDNA-guided vs 92.4% standard (difference 1.1 percentage points; 95% CI -4.1 to 6.2).	Personalized tumor-informed ctDNA assay tracking patient-specific mutations.
Tie, J. et al., 2025 [30]	https://doi.org/10.1038/s41591-025-03579-w	Five-year follow-up of randomized DYNAMIC trial	DYNAMIC stage II colon cancer cohort; median follow-up 59.7 months	ctDNA-guided versus standard management	Tumor-informed ctDNA; expanded EOT assay assessed ~29 tumor-derived mutations per patient	Not assessed	Not assessed	Postoperative mutant molecules/mL assessed; formal sensitivity/specificity NR.	Among treated ctDNA-positive patients, EOT clearance occurred in 35/40 (87.5%). Five-year recurrence-free probability was 97% with clearance vs 0% with persistence.	Five-year outcome assessment.	Higher than median postoperative mutant molecules/mL was associated with worse 5-year RFS: HR 10.62 (95% CI NR). Five-year RFS 88%	Long-term follow-up supported reduced ACT use without compromising 5-year RFS or OS.	Personalized tumor-informed ctDNA assays; expanded mutation panel for EOT clearance analysis.

											ctDNA-guided vs 87% standard; 5-year OS 93.8% vs 93.3% (HR 1.05; P=0.887).		
Tie, J. et al., 2025 [31]	https://doi.org/10.1038/s41591-025-04030-w	Randomized phase 2/3 DYNAMIC-III trial	968 evaluable patients with stage III colon cancer; Australia and Canada	ctDNA-guided escalation/de-escalation versus standard management	Tumor-informed personalized ctDNA assay	Not assessed	Not assessed	ctDNA assessed 5-6 weeks after surgery. Three-year RFS: 87% ctDNA-negative vs 49% ctDNA-positive.	Persistent ctDNA after treatment: 3-year RFS 14% vs 79% with clearance.	Serial ctDNA clearance and recurrence follow-up.	Higher ctDNA burden correlated with recurrence risk; 3-year RFS ranged from 77% to 23% across burden quartiles.	In ctDNA-negative patients, de-escalation reduced oxaliplatin use (34.8% vs 88.6%) and hospitalizations (8.5% vs 13.2%) but did not meet RFS noninferiority (85.3% vs 88.1%). In ctDNA-positive patients, escalation did not improve 2-year RFS (51% vs 61%).	Personalized tumor-informed ctDNA assay.
